# Generation of PDGFRα^+^ Cardioblasts from Pluripotent Stem Cells

**DOI:** 10.1038/srep41840

**Published:** 2017-02-06

**Authors:** Seon Pyo Hong, Sukhyun Song, Sung Woo Cho, Seungjoo Lee, Bong Ihn Koh, Hosung Bae, Kyun Hoo Kim, Jin-Sung Park, Hyo-Sang Do, Ilkyun Im, Hye Jin Heo, Tae Hee Ko, Jae-Hyeong Park, Jae Boum Youm, Seong-Jin Kim, Injune Kim, Jin Han, Yong-Mahn Han, Gou Young Koh

**Affiliations:** 1 Biomedical Science and Engineering Interdisciplinary Program, Korea Advanced Institute of Science and Technology (KAIST), Daejeon, Korea; 2 Center for Vascular Research, Institute for Basic Science (IBS), Daejeon, Korea; 3 Graduate School of Medical Science and Engineering, Korea Advanced Institute of Science and Technology (KAIST), Daejeon, Korea; 4 Department of Biological Sciences, Korea Advanced Institute of Science and Technology (KAIST), Daejeon, Korea; 5 Cardiovascular and Metabolic Disease Center, Department of Physiology, College of Medicine, Inje University, Busan, Korea; 6 Department of Cardiology in Internal Medicine, School of Medicine, Chungnam National University, Daejeon, Korea; 7 CHA Cancer Institute, Department of Biomedical Science, CHA University, Seoul, Korea

## Abstract

Isolating actively proliferating cardioblasts is the first crucial step for cardiac regeneration through cell implantation. However, the origin and identity of putative cardioblasts are still unclear. Here, we uncover a novel class of cardiac lineage cells, PDGFRα^+^Flk1^−^ cardioblasts (PCBs), from mouse and human pluripotent stem cells induced using CsAYTE, a combination of the small molecules Cyclosporin A, the rho-associated coiled-coil kinase inhibitor Y27632, the antioxidant Trolox, and the ALK5 inhibitor EW7197. This novel population of actively proliferating cells is cardiac lineage–committed but in a morphologically and functionally immature state compared to mature cardiomyocytes. Most important, most of CsAYTE-induced PCBs spontaneously differentiated into functional αMHC^+^ cardiomyocytes (M^+^CMs) and could be a potential cellular resource for cardiac regeneration.

Cardiovascular diseases remain a leading cause of mortality worldwide, with death arising from the inability of cardiomyocytes to regenerate after myocardial injury. In this aspect, cardiac lineage cells (CLCs) from pluripotent stem cells (PSCs) have become the most attractive cellular resource underlying an unprecedented strategy in cell-based therapy to rescue damaged hearts^1^,^2^,^3^. Recently, major advances have been achieved in generation of cardiac precursor cells from human PSCs with high efficiency, and are becoming a reliable and clinically applicable cellular resource for cardiac regeneration^4^,^5^,^6^. For instances, Burridge *et al*.^4^ successfully produced cardiomyocytes with up to 95% purity from human induced PSCs (iPSCs) with relatively high efficiency using a chemically defined medium. Moreover, differentiating cardiomyocytes derived from certain human PSCs can be achieved up to a clinical scale, and these cells can be electromechanically coupled with host cardiomyocytes in a non-human primate model of myocardial ischemia^6^. Nevertheless, obtaining a sufficient amount of cardiac precursor cells or differentiated cardiomyocytes from PSCs is the most important challenge.

Cardiac specification and further differentiation processes from embryonic stem cells (ESCs) are quite complex and delicate; thus, each individual step of the induction, specification, and differentiation needs to be finely regulated^7^. For example, Flk1^+^ mesodermal precursor cells (MPCs) derived from differentiating PSCs were identified as cardiovascular progenitors that can give rise to cardiac, endothelial, hematopoietic, and mural lineage cells via multiple different signaling pathways both *in vitro* and *in vivo*^8^,^9^,^10^,^11^,^12^. Various molecules have been tested to restrict the differentiation of PSCs into cardiac lineages, most of which are related to signaling pathways, such as bone morphogenetic protein, transforming growth factor, activin, nodal, Wnt, rho-associated coiled-coil kinase (ROCK), and fibroblast growth factor^7^,^13^. However, each signaling pathway has no stringent role solely for cardiac specification and differentiation of mouse PSCs^7^, while temporal inhibition of canonical Wnt signaling is reported as a key modulator for the efficient cardiac differentiation from human PSC^14^.

We previously reported that a combination of Cyclosporin A (CsA) and antioxidants synergistically promotes cardiac differentiation from Flk1^+^ MPCs by modulating the mitochondrial permeability transition pore (mPTP) and redox signaling^15^. Here, we screened for various signaling modulators and established a novel, simple, and efficient method for cardiac lineage specification from mouse PSC-derived Flk1^+^ MPCs using a combination of four specific modulators: CsA, the ROCK inhibitor Y27632, the antioxidant Trolox, and the activin A receptor type II-like kinase (ALK5) inhibitor EW7197 (referred to collectively here as CsAYTE). Of special note, CsAYTE strongly induced the commitment of Flk1^+^ MPCs into PDGFRα^+^Flk1^−^ cardioblasts (referred to as PCBs), a novel subpopulation of CLCs with distinct features. We showed that CsAYTE-induced PCBs not only could proliferate but also could spontaneously further differentiate into functional cardiomyocytes with high efficiency, which can be a novel cellular resource for cardiac regeneration.

## Results

### CsAYTE promotes commitment of PSCs into CLCs

**Figure 1:**
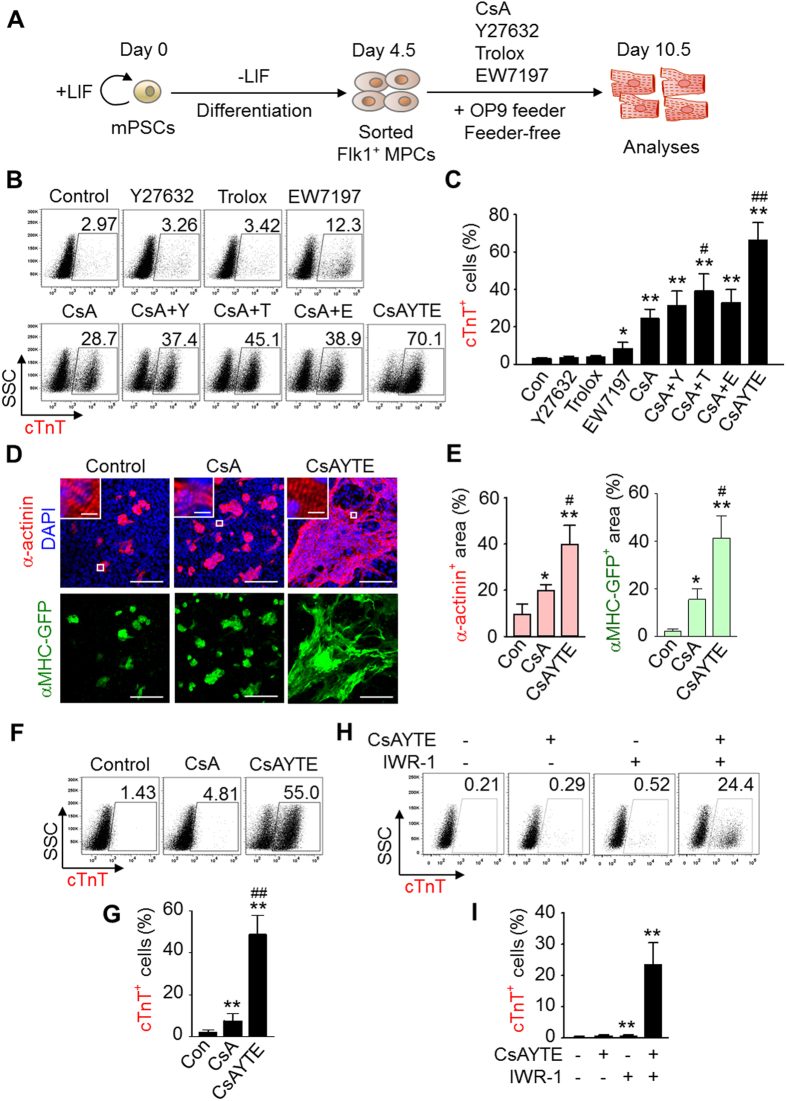
CsAYTE promotes commitment of PSCs into CLCs. (**A**) Protocol for the commitment of Flk1^+^ MPCs into CLCs induced by four specific modulators in an OP9 co-culture system. LIF, Leukemia inhibitory factor. (**B** and **C**) Representative FACS analysis and the percentage of mouse ESC-derived cTnT^+^ cells incubated with the indicated agents. Con, control vehicle; Y, Y27632 (10 μM); T, Trolox (400 μM); E, EW7197 (1 μg/mL); CsA (3 μg/mL). Each group, n = 4. (**D** and **E**) Images displaying α-actinin^+^ cells, DAPI^+^ nuclei and αMHC-GFP^+^ cells (Scale bars, 100 μm), Inset: High resolution confocal image indicating sarcomeric structure (Scale bars, 5 μm), and comparison of α-actinin^+^ area (%) and αMHC-GFP^+^ area (%). Each group, n = 3–4. (**F** and **G**) Representative FACS analysis and percentage of mouse iPSC-derived cTnT^+^ cells grown in OP9 co-culture. Each group, n = 4. In C, E, G graphs, *p < 0.05 and **p < 0.01 versus Con; ^#^*p* < 0.05 and ^##^*p* < 0.01 versus CsA. (**H** and **I**) Representative FACS analysis and percentage of mouse ESC-derived cTnT^+^ cells grown in a feeder-free culture with or without Wnt signaling inhibitor IWR-1 (2 μM). Each group, n = 5. **p < 0.01 versus Control.

Our previous study showed that CsA treatment increases the commitment of PSCs into CLCs by about 10-fold by activating mitochondrial oxidative metabolism mediated through mPTP inhibition^15^. Under this condition, addition of antioxidants further augmented this CsA-induced CLC commitment^15^. Because the inhibition of ROCK or ALK5 signaling contributes to cardiomyogenesis^16^,^17^,^18^,^19^, we hypothesized that a combinatorial treatment with all four specific modulators could synergistically promote commitment of PSCs into CLCs. For monitoring and tracing CLC commitment, we used EMG7 embryonic stem cells (ESCs), which have a transgene consisting of cardiac-specific α myosin heavy chain (αMHC) promoter–driven enhanced green fluorescent protein (GFP); in addition, we screened for the cardiac-specific markers cardiac troponin T (cTnT) and α-actinin in differentiating PSCs. At day 4.5 after mesodermal induction without leukemia inhibitory factor in ESCs, Flk1^+^ MPCs were sorted and plated onto a mitomycin-c–treated OP9 feeder-cell layer or feeder-cell layer-free dish, and the four specific modulators were added to the differentiation medium. CLC commitment and differentiation were analyzed at day 10.5 (Fig. 1A).

As the four specific modulators, we used CsA for mPTP inhibition^15^, Y27632 for ROCK inhibition^11^, Trolox as an antioxidant^15^, and EW7197 (interchangeable with TEW7197) (Fig. S1A) for ALK5 inhibition^20^. Dose optimization was determined by the relative total cell number and percentage of cTnT^+^ cells; the optimal dose of each modulator was as follows: 3 μg/mL of CsA; 10 μM of Y27632; 400 μM of Trolox; and 1 μg/mL of EW7197 (Fig. S1B–E). The optimal dose of CsA, Y27632, Trolox, or EW7197 induced Flk1^+^ MPC differentiation into cTnT^+^ cells at average rates ranging from 3.78% to 24.5%, and the combination of Y27632, Trolox, or EW7197 with CsA further promoted differentiation on average from 31.3% to 39.3% (Fig. 1B,C). However, the combination of all four modulators, i.e., CsAYTE, strikingly promoted Flk1^+^ MPC differentiation into cTnT^+^ cells at a rate of ~70% (Fig. 1B,C). Accordingly, CsAYTE profoundly increased the area of self-beating cells (Movie S1), the area of α-actinin^+^ cells up to 39.9%, and the area of αMHC-GFP^+^ cells up to 41.5% (Fig. 1D,E). Similarly, CsAYTE also increased mouse iPSC-derived Flk1^+^ MPC differentiation into cTnT^+^ cells at a rate of 50–55% (Fig. 1F,G).

In contrast, in a feeder-free culture condition, CsAYTE did not promote differentiation of Flk1^+^ MPCs into cTnT^+^ cells (Fig. 1H,I), implying that the secretory factors from the feeder cells could be critical in CsAYTE-induced CLC commitment and cardiac differentiation. Given that Wnt signaling inhibition effectively induces cardiac differentiation^12^, we added an optimal dose of the Wnt inhibitor IWR-1 (2 μM) and found that it promoted cardiac differentiation up to 25.3% (Fig. 1H,I). These data confirm that secretory factors including endogenous Wnt signaling inhibitor from the feeder cells are critical for the CsAYTE-induced CLC commitment and cardiac differentiation, which warrants further investigation. Also important, another mPTP inhibitor NIM811, ROCK inhibitor RKI1447, antioxidant N-acetyl-L-cysteine, and ALK5 inhibitor SB431542 could replace CsAYTE to induce the synergistic CLC commitment effect. However, the combination of CsA with other signaling modulators, such as inhibitor of PI3-kinase, MEK, ERK, PKA, PKC, PKG, mTOR, GSK3β, notch, AMPK, MLC kinase, or PPARα, either inhibited or did not affect Flk1^+^ MPC differentiation into cTnT^+^ cells (Fig. S1F). Thus, CsAYTE is a strong combination of inducers to generate CLCs from MPCs with significantly higher efficiency.

### CsAYTE induces Flk1^+^ MPC differentiation into PDGFRα^+^Flk1^−^ cardioblasts

**Figure 2:**
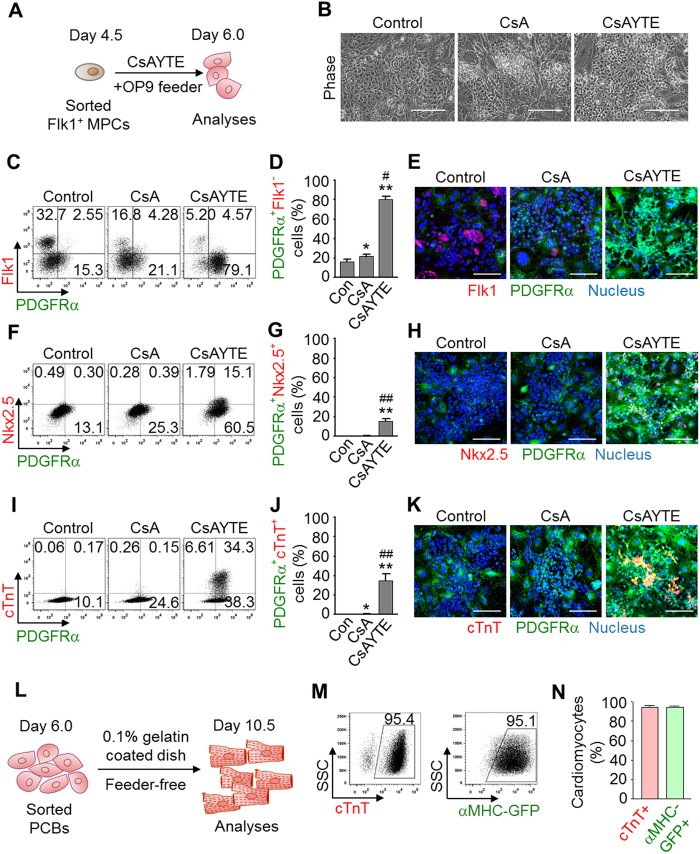
CsAYTE generates PCBs that eventually differentiate into cardiomyocytes. (**A**) Protocol to generate PCBs from Flk1^+^ MPC by CsAYTE stimulation and subsequent analyses. (**B**) Phase-contrast images showing differentiating Flk1^+^ MPCs at day 6.0 incubated with control vehicle (Control), CsA, and CsAYTE. Scale bars, 100 μm. (**C**–**K**) Representative FACS analyses, quantifications, and images of PDGFRα^+^Flk1^−^ PCBs, PDGFRα^+^ Nkx2.5^+^ cells, and PDGFRα^+^ cTnT^+^ cells differentiated from Flk1^+^ MPCs at day 6.0 incubated with Control, CsA, and CsAYTE (Scale bars, 100 μm). Each group, n = 3–6. *p < 0.05 and **p < 0.01 versus Con; ^#^*p* < 0.05 and ^##^*p* < 0.01 versus CsA. (**L**) Protocol for analyses of PCB-derived cardiomyocyte differentiation in a feeder-free culture. (**M** and **N**) Representative FACS analyses and percentages of PCBs-derived cTnT^+^ and αMHC-GFP^+^ cardiomyocytes grown in feeder-free culture. Each group, n = 4–5.

Of special note, during the process of differentiating Flk1^+^ MPCs into CLCs (Fig. 2A), the morphology of cells changed homogeneously to a small and round shape within a day after CsAYTE treatment but did not appear to change with control vehicle or CsA alone (Fig. 2B). These morphologically homogeneous cells rapidly expanded in small colonies until they came into contact with other expanding cells from adjacent colonies (Movie S2), and started to beat synchronously and express αMHC-GFP at day 7.5–8.0 (Fig. S2A,B) throughout the course of differentiation. Therefore, this homogeneous cell population exhibited the hallmark features of early cardiac precursor cells, and we defined them as cardioblasts.

To further identify and characterize this cell population based on surface marker expression, we screened for several previously reported cardiovascular progenitor markers, such as Flk1, PDGFRα, PDGFRβ, CXCR4, ALCAM, and c-kit^12^,^21^,^22^,^23^,^24^,^25^. Among them, only PDGFRα was expressed in most of these putative cardioblasts while the expression of Flk1 was abruptly reduced within 36 h (Fig. 2C–E). Thus, within 36 h, CsAYTE strongly induced Flk1^+^ MPC differentiation into and consequential expansion of PDGFRα^+^ Flk1^−^ cardioblasts (hereafter designated as PCBs) in OP9 feeder cell culture up to 80% and 70% from both mouse ESCs and iPSC-derived Flk1^+^ MPCs, respectively; control vehicle and CsA treatment alone induced 16% and 22% differentiation into PCBs, respectively (Fig. 2C–E and S2C–E). Of importance, at day 6 under CsAYTE stimulation, ~15% of PCBs co-expressed Nkx2.5, a representative cardiac transcription factor, but ~35% of PCBs already expressed cTnT protein (Fig. 2F–K). In contrast, few or no Nkx2.5^+^ or cTnT^+^ cells were observed with control or CsA stimulation at day 6 (Fig. 2F–K). These results indicate that PCBs are composed of early cardioblasts and differentiating cardiomyoblast intermediates.

To identify the most effective modulator for PCB commitment, we further investigated the PCB commitment efficiency of each modulator in CsAYTE at day 6. Among the four modulators, EW7197 most effectively (51%) induced Flk1^+^ MPC differentiation into PCBs whereas CsA, Y27632 and Trolox were less effective (18–27%). These data indicate that inhibition of ALK5 signaling pathways is crucial for the induction of Flk1^+^ MPC differentiation into PCBs (Fig. S2F,G). Nevertheless, PCBs were not induced in a feeder-free culture condition, implying that secretory factors from the feeder cells could be critical in CsAYTE-induced the PCB commitment. In contrast, PDGFRα expression was not observed in CD31^+^ endothelial cells or CD41^+^ early hematopoietic cells regardless of the modulator used. In fact, CsAYTE markedly reduced differentiation from Flk1^+^ MPCs into CD144^+^ CD31^+^ endothelial cells and CD41^+^ early hematopoietic cells to less than 1% (Fig. S2H–M).

To test whether continuous CsAYTE treatment or OP9 feeder cells are required for complete differentiation of PCBs into cardiomyocytes, we sorted PCBs at day 6 and incubated them on 0.1% gelatin-coated dishes at a high-density culture condition (equal or more than 5 × 10^5^ cells/cm^2^) for the next 4.5 days (Fig. 2L). More than 95% of the sorted PCBs spontaneously differentiated into cTnT^+^ and αMHC-GFP^+^ cells without CsAYTE treatment in OP9 feeder cell-free conditions (Fig. 2M,N). These data indicate that PCBs require a certain cell-to-cell contact rather than continuous CsAYTE-induced signaling or secretory factors from the feeder cells to differentiate into mature cardiomyocytes, once they are committed to the cardiac lineage as cardioblasts. In addition, to demonstrate whether PCBs are restricted to differentiation into cardiomyocytes even in non-cardiomyocyte differentiation conditions, for example supplementation with endothelial cell or vascular smooth muscle cell differentiation growth factor, we incubated PCBs in the differentiation medium under VEGF-A (200 ng/ml) or PDGF-BB (50 ng/ml) stimulation in OP9 feeder cell-free conditions. Exogenous VEGF-A or PDGF-BB did not significantly change the population of cTnT^+^ cells (Fig. S3A–C). These results indicated that the differentiation potential of PCBs is limited to cardiomyocytes even under non-cardiac differentiation conditions.

We also investigated whether the enrichment of PDGFRα expression in PCBs plays a functional role in cardiac differentiation via PDGF–PDGFRα signaling. However, treating PCBs with PDGF ligands, PDGF-AA, -BB, -AB, -CC, and -DD, or with a specific PDGFRα inhibitor crenolanib did not significantly change the population of cTnT^+^ cells (Fig. S4A–E). Moreover, no significant changes were found in cardiac differentiation by depletion of PDGFRα mRNA expression up to ~60% in MPCs and PCBs (Fig. S4F–J). Thus, PDGF–PDGFRα signaling is overridden by other dominant commitment processes for PCBs or PDGFRα is merely a surface marker for PCBs, rather than an active, functional receptor regulating cellular responses.

### Rapid amplification and differentiation of Flk1^+^ MPCs into PCBs by CsAYTE

**Figure 3:**
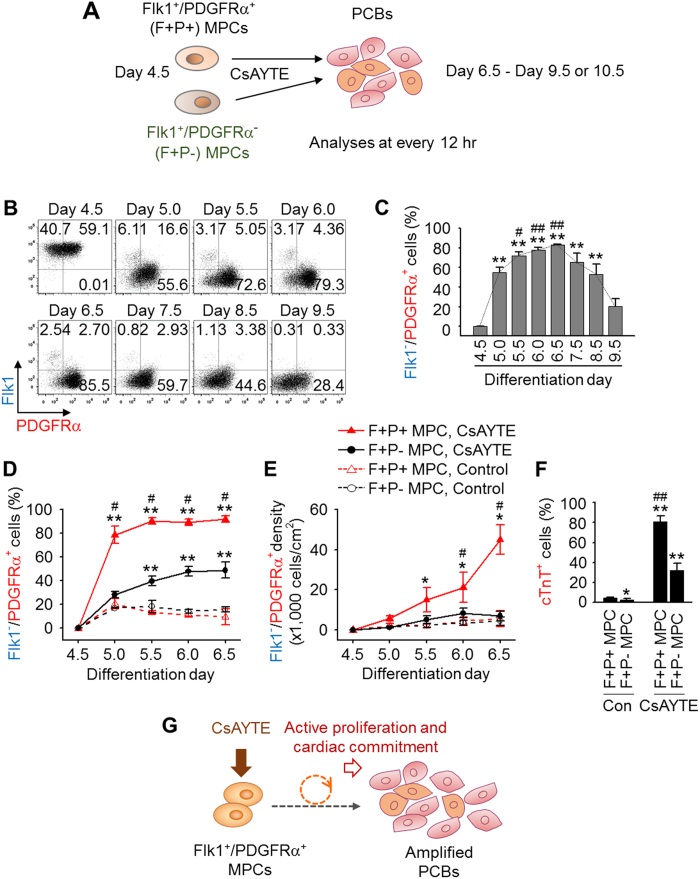
Rapid conversion of Flk1^+^ MPCs into PCBs by CsAYTE. (**A**) Protocol for analyses of PCB generation from Flk1^+^ MPCs. (**B** and **C**) Representative FACS analyses and quantifications of differentiating Flk1^−^/PDGFRα^+^ PCBs derived from Flk1^+^ MPCs incubated with CsAYTE at every 12 h from day 4.5 for 5 days. Each group, n = 3–5. **p < 0.01 versus day 4.5; ^#^*p* < 0.05 and ^##^*p* < 0.01 versus day 5.0. (**D**–**F**) Flk1^+^ MPCs were divided into Flk1^+^/PDGFRα^+^ (F+P+) and Flk1^+^/PDGFRα^−^ (F+P−) MPCs, incubated with Control and CsAYTE, analyzed for populations and densities of Flk1^−^/PDGFRα^+^ (F−P+) PCBs at every 12 h from day 4.5 for 2 days, and percentages of cTnT^+^ cardiomyocytes were assessed at day 10.5. Each group, n = 5. *p < 0.05 and **p < 0.01 versus F+P+MPCs, Control at each time point; ^#^*p* < 0.05 and ^##^*p* < 0.01 versus F+P− MPCs, CsAYTE at each time point. (**G**) Schematic diagram depicting how CsAYTE selectively promotes the commitment and differentiation of Flk1^+^ MPCs into PCBs, while it simultaneously and robustly expands number of PCBs.

To investigate how CsAYTE expanded PCBs robustly within a short period, we isolated Flk1^+^ MPCs at day 4.5, treated them with CsAYTE, and tracked changes in the expression of Flk1 and PDGFRα every 12 h for 5 days (Fig. 3A). Two populations, Flk1^+^/PDGFRα^+^ (F+P+) MPCs (~55%) and Flk1^+^/PDGFRα^−^ (F+P−) MPCs (~45%), were present in the isolated Flk1^+^ MPCs at day 4.5 (Fig. 3B and S5A,B). We first noted that most of these cells (>90%) lost Flk1 expression within 24 h and continued to gradually decrease over time under CsAYTE stimulation until virtually no cells expressed Flk1 (Fig. 3B and S5B). We then focused on tracing the Flk1^−^/PDGFRα^+^ (F−P+) cells, which we have previously defined as PCBs. The population of F−P+ PCBs was sharply increased from 0% to 55% after 12 h, gradually increased to 83% until day 6.5, and then rapidly decreased over the following 3 days (Fig. 3B,C). Further detailed analysis revealed that under CsAYTE stimulation, most F+P+ MPCs were rapidly differentiated into F−P+ PCBs within 12 h with higher proliferation activity while ~40–50% of F+P− MPCs were differentiated into F−P+ PCBs over 2 days with much less proliferation activity (Fig. 3D,E and S5B). In comparison, only small numbers (<20%) of F+P+ MPCs and F+P− MPCs were differentiated into F−P+ PCBs over 2 days, with low proliferation activity under control conditions (Fig. 3D,E and S5B). Moreover, F+P+ MPCs showed a higher cardiac differentiation ability than F+P− MPCs under CsAYTE stimulation, and both had little (<5%) cardiac differentiation ability under control conditions (Fig. 3F). These data indicate that F+P+ MPCs are the major source of PCBs and subsequent cTnT^+^ cardiomyocytes under CsAYTE stimulation, which is similar to a previous report^26^. Thus, CsAYTE selectively promotes the commitment and differentiation of Flk1^+^ MPCs into CLCs while inhibiting their differentiation into endothelial or hematopoietic lineage cells, as well as simultaneously and robustly expanding the number of CLCs (Fig. 3G). We define this process as cardiac specification and generation of cardioblasts.

To investigate whether PCBs could expand while keeping their differentiation potential over a long period, we tried to expand PCBs following recent studies^27^,^28^ for long-term expansion of cardiovascular progenitor cells. We sorted PCBs at day 6.0, seeded onto a 0.1% gelatin-coated culture plate at a low-density (6.5 × 10^4^ cells/cm^2^), and incubated them with either control vehicle, CsAYTE, BIO (2.5 μM) + LIF (10^3^ units/ml), or BACS (5 ng/ml BMP4, 10 ng/ml Activin A, 3 μM CHIR99021, and 2 μM SU5402) (Fig. S6A). The cells expanded from PCBs treated only with BIO and LIF kept PDGFRα expression (95.2 ± 2.4%) (Fig. S6B,C). However, these expanded cells could not differentiate into cardiomyocytes (cTnT^+^ cells, 2.4 ± 1.2%) when the expanded cells were incubated at a high-density (5 × 10^5^ cells/cm^2^) in a differentiation medium without BIO and LIF (Fig. S6D,E). Consequentially, CsAYTE could efficiently induce and expand PCBs but only for a short period, and they lost their proliferative potential after fully differentiating cardiomyocytes.

### Histone modification during PCB generation

To investigate the molecular mechanisms of PCB generation from mouse ESC-derived Flk1^+^ MPCs, we evaluated histone modification and DNA methylation in the promoter regions of mesodermal and cardiac transcription factors. We analyzed histone 3 lysine 27 trimethylation (H3K27me3), which is a characteristic of inactive chromatin, and H3K4me1, H3K4me3, and H3K9 acetylation (H3K9ac), which all characterizes active promoters. In a chromatin immunoprecipitation assay, mesodermal genes such as *brachyury* and *mesp1* showed a decrease in H3K4me3 at their promoters in PCBs compared with Flk1^+^ MPCs (Fig. S7A,B). Of note, among the cardiac transcription factors, *meis1* and *tbx5* showed an enrichment of H3K4me1, H3K4me3, and H3K9ac at their promoters in PCBs compared with Flk1^+^ MPCs (Fig. S7C,D). However, *nkx2.5* and *gata4* did not show any substantial changes in histone marks (Fig. S7E,F). Furthermore, DNA methylation of each gene at its promoter was not significantly changed in PCBs compared with Flk1^+^ MPCs (Fig. S7G). These results indicate that activation of chromatin by histone modification at promoters of *meis1* and *tbx5* contributes to cardioblast commitment from Flk1^+^ MPC.

### Human PSCs differentiate into PCBs under CsAYTE stimulation

**Figure 4:**
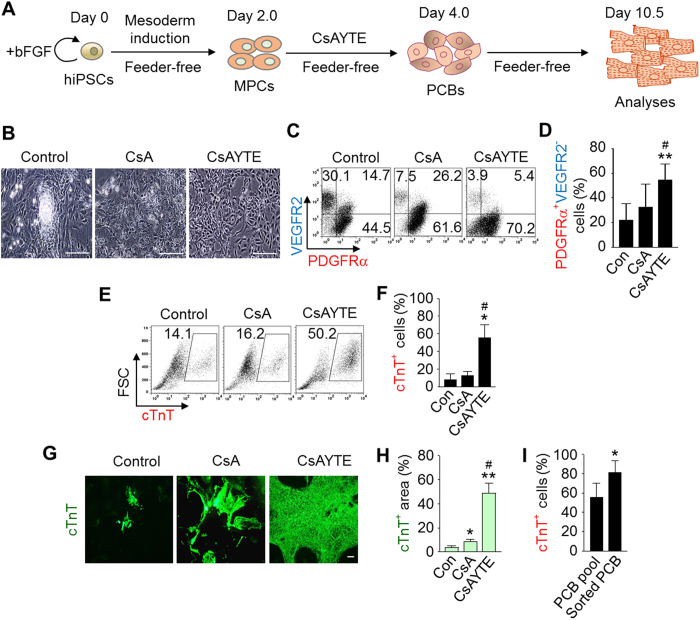
Human PSCs differentiates into PCBs under CsAYTE stimulation. (**A**) Protocol to generate PCBs in human iPSCs by CsAYTE stimulation. (**B**) Phase-contrast images showing differentiating MPCs at day 4.0 from human iPSCs incubated with Control, CsA, and CsAYTE. Scale bars, 100 μm. (**C** and **D**) Representative FACS analysis and quantification of PDGFRα^+^ VEGFR2^−^ cells at day 6.0 from human iPSCs incubated with Control, CsA, and CsAYTE. Each group, n = 6. (**E** and **F**) Representative FACS analysis and percentage of human iPSC-derived cTnT^+^ cells grown in feeder-free culture at day 10.5. Each group, n = 3–4. (**G** and **H**) Images displaying human iPSC-derived cTnT^+^ cells at day 10.5 and the quantification analysis of cTnT^+^ area (%). Each group, n = 3. In all graphs, *p < 0.05 and **p < 0.01 versus Con; ^#^*p* < 0.05 versus CsA. Scale bars, 100 μm. (**I**) Percentage of cTnT^+^ cells at day 10.5 after sorting of PCBs at day 4.0. Each group, n = 4. *p < 0.05 versus PCB pool.

To recapitulate the differentiation process into PCBs with human PSCs, we treated MPCs derived from human iPSCs with CsAYTE under a feeder-free condition (Fig. 4A). Similarly, CsAYTE not only changed the MPCs to a homogeneous morphology within 48 h (Fig. 4B) but also enhanced their differentiation into PDGFRα^+^ VEGFR2^−^ cardioblasts up to 55% (Fig. 4C,D). Moreover, CsAYTE enhanced the representation of cTnT^+^ cells to 55.6% and the area of cTnT^+^ cells to 48.8% in human iPSCs by day 10.5 while the proportions of cTnT^+^ cells were 7.8% and 12.3%, and the areas of cTnT^+^ cells were 3.56% and 8.38% in the control vehicle and CsA alone groups, respectively (Fig. 4E–H). Thus, CsAYTE could generate PCBs from human PSCs and subsequently promote their cardiac differentiation. Furthermore, sorted PDGFRα^+^ VEGFR2^−^ cardioblasts at day 4 differentiated into cTnT^+^ cardiomyocytes by ~80% at day 10.5 (Fig. 4I), confirming that the human cardioblasts also possess cardiac progenitor potential.

### PCBs are proliferating cardiac lineage–committed cells in a morphologically and functionally immature state

**Figure 5:**
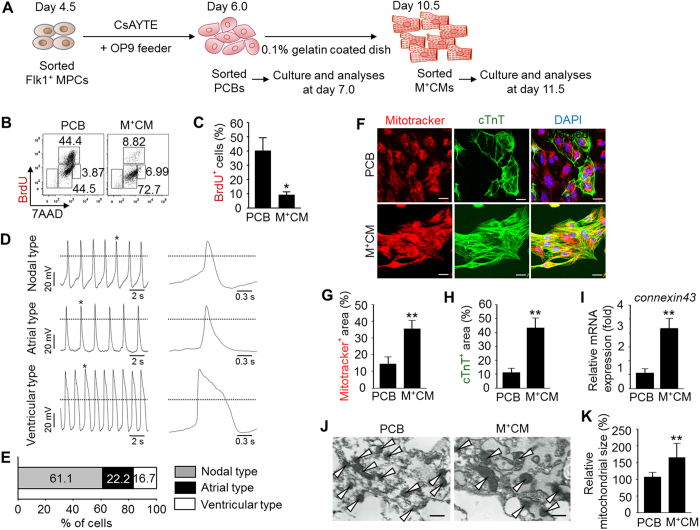
PCBs are in a morphologically and functionally immature state. (**A**) Protocol for generation and analyses of PCBs and αMHC-GFP^+^ cardiomyocytes (M^+^CMs). (**B** and **C**) Representative FACS analysis of BrdU incorporation and the percentage of BrdU^+^ cells in PCBs and M^+^CMs. Each group, n = 3. (**D** and **E**) 3 different types (nodal, atrial, and ventricular type) of action potentials and percentile distribution in M^+^CMs. Each group, n = 3. Dotted lines indicate zero voltage level. (**F**–**H**) Images showing Mitotracker^+^ mitochondria. cTnT^+^ sarcomere and DAPI^+^ nuclei, and comparisons of Mitotracker^+^ and cTnT^+^ areas in PCBs and M^+^CMs. Scale bars, 20 μm. Each group, n = 6. (**I**) Relative mRNA expression levels of *connexin43* gap junction in PCBs and M^+^CMs. Each group, n = 3. (**J** and **K**) Transmission electron microscope images showing the mitochondrial morphology and cristae (white arrow heads) and quantification of mitochondrial size in PCBs and M^+^CMs. Scale bars, 500 nm. Each group, n = 8. In all graphs, *p < 0.05 and **p < 0.01 versus PCB.

The degree of cardiomyocyte differentiation can be characterized not only by the expression patterns of cardiac-specific markers including αMHC, cTnT, and α-actinin but also by functional attributes, such as firing action potentials and global transcriptome analysis^29^. To characterize this novel PCB population, we investigated the properties of PCBs and compared them with PCB-derived differentiated αMHC-GFP^+^ cardiomyocytes (hereafter referred to as M^+^CMs). First, to gain insight into the cellular and functional properties of PCBs and M^+^CMs, cells were sorted at days 6.0 and 10.5, plated onto 0.1% gelatin-coated dishes, and analyzed and compared after one day (Fig. 5A). As anticipated, the PCB population had a relatively higher proportion (40.1%) of BrdU^+^ proliferating cells than M^+^CMs (9.0%) (Fig. 5B,C). On the other hand, we noted that PCBs did not show any notable electrical recordings in whole-cell patch clamp analysis whereas M^+^CMs showed constant and robust firing of spontaneous nodal, atrial, and ventricular action potentials and ion currents, such as delayed rectifier K^+^ current (I_K_), voltage gated Na^+^ current (I_Na_), and T-type Ca^2+^ currents (I_CaT_) (Fig. 5D,E and S8A–C). These ion currents were inhibited by ion channel blockades, such as the potassium channel blocker tetraethylammonium (20 mM), sodium channel blocker tetrodotoxin (1 μM), and calcium channel blocker mibefradil (1 μM) (Fig. S8A–C). These data clearly indicate that M^+^CMs, not PCBs, have electrical properties and function. Of note, despite the lack of electrical properties and function, Flk1^+^ MPCs and PCBs still expressed ion channels including Kir 2.1, Nav 1.3, and Cav 3.2, although they expressed them less than did M^+^CMs (Fig. S8D). These data suggest that the ion channels and contractile structures are not yet properly coupled in the PCBs while they are coupled relatively well in M^+^CMs. Furthermore, compared with M^+^CMs, PCBs had 21% and 32% less Mitotracker^+^ mitochondria and cTnT^+^ sarcomere areas, respectively, and the mRNA expression level of *connexin43* gap junctions was 44% less (Fig. 5F–I). Transmission electron microscope images also showed under-developed (or immature) mitochondrial cristae and smaller mitochondrial sizes (white arrowheads) in PCBs (Fig. 5J,K).

**Figure 6:**
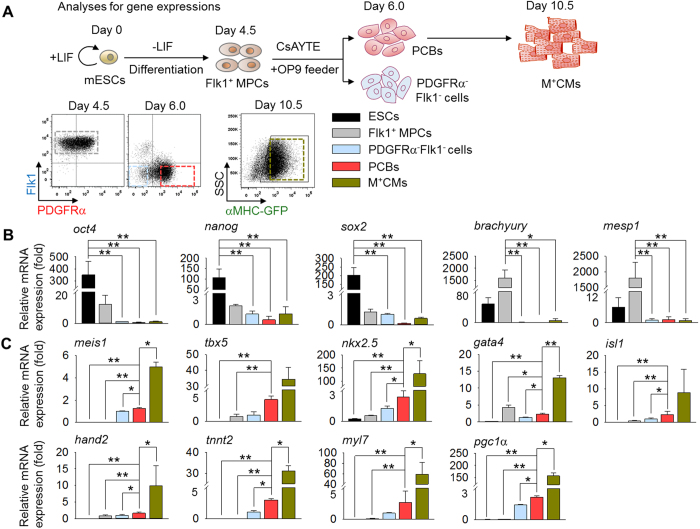
PCBs are at an intermediate state between MPC and differentiated cardiomyocytes. (**A**) Protocol for gene expression analyses of ESCs, Flk1^+^ MPCs, PDGFRα^-^Flk1^−^ cells, PCBs, and M^+^CMs. (**B** and **C**) Relative mRNA expression levels of pluripotency- (*oct4, nanog* and *sox2*), mesoderm- (*brachyury, mesp1*), cardiac transcription factor- (*meis1, tbx5, nkx2.5, gata4, isl1* and *hand2*), cardiac sarcomere protein- (*tnnt2* and *myl7*), and mitochondrial biogenesis- (*pgc1α*) related genes in the indicated cells. All values are relative to that of PDGFRα^−^Flk1^−^ cells. In all graphs, *p < 0.05 and **p < 0.01 versus indicated cells.

To further delineate the molecular properties of PCBs, we sorted cells, analyzed cardiac-related gene expression, and compared them with ESCs, Flk1^+^ MPCs, PDGFRα^−^Flk1^−^ cells, and M^+^CMs (Fig. 6A). PCBs did not express pluripotent genes, such as *oct4, nanog*, and *sox2*, or mesodermal genes, including *mesp1* and *brachyury* (Fig. 6B). However, expression levels of cardiac-related transcription factors, such as *meis1, tbx5, nkx2.5*, and *isl1*, but not *gata4* and *hand2*, were increased compared with more primitive populations while showing lower levels in contrast to further differentiated M^+^CMs. Cardiac-specific genes, including *tnnt2* and *myl7*, and mitochondrial biogenesis markers, such as *pgc1α*, also showed similar patterns (Fig. 6C). These data suggest that PCBs are in an intermediate state between MPCs and differentiated cardiomyocytes.

Finally, to elucidate the genome-wide characteristics of PCBs, microarray analysis was performed and results compared with those of the spontaneously formed PCBs that were obtained without CsAYTE incubation (hereafter referred to as sfPCBs), Flk1^+^ MPCs, and M^+^CMs (Fig. S9A,B). Comparison of PCBs and Flk1^+^ MPCs identified 558 differently (≥30 fold) expressed transcripts. Gene ontology analysis also showed that PCBs highly expressed genes belonging to chemical and cytokine stimulus, cardiovascular system development, and cell adhesion and proliferation compared with Flk1^+^ MPCs (Fig. S9C). Moreover, comparison of PCBs and sfPCBs at day 5.5 identified 163 differently (≥30 fold) expressed transcripts. Gene ontology analysis revealed a significant increase in expression of genes related to heart and muscle development in PCBs compared with sfPCBs (Fig. S9D). Of note, gene expression profiles and ontology of M^+^CMs revealed a robust upregulation of genes related to mitochondrial function and metabolism and ion channel activity compared with PCBs (Fig. S9E). Collectively, these results indicate that PCBs can be characterized as proliferating cardiac lineage–committed cells, which are still in a morphologically and functionally immature state compared with differentiated cardiomyocytes.

## Discussion

Here, we report a novel subpopulation of CLCs featured as PDGFRα^+^Flk1^−^ cardioblasts, which can be copiously generated from MPCs through robust cardiac commitment and specification with CsAYTE, a combination of four specific modulators. These cardioblasts actively proliferate while exhibiting hallmark cardiac features, such as expression of cardiac-specific genes and contractile proteins, and they spontaneously differentiate into mature cardiomyocytes with relatively high efficiency. Thus, CsAYTE is a powerful combination for generating an ample amount of cardioblasts by actively and simultaneously driving proliferation and cardiac commitment to subsequently yield functional cardiomyocytes that can be used for the regeneration of damaged hearts.

The original concept of cardioblasts in mammals has been established by the discovery of isl1^+^ cells in embryonic and postnatal hearts^30^, but a subsequent study^31^ indicated that these isl1^+^ cells are cardiovascular progenitor cells that give rise to cardiomyocytes, smooth muscle cells, and endothelial cells. Another recent study^32^ indicated that HopX^+^ cells in embryonic hearts could be true cardioblasts that are differentiated only into cardiomyocytes. However, the detailed identity and features of HopX^+^ cardioblasts warrant more study. Moreover, it remains to study what features these PDGFRα^+^ cardioblasts share with isl1^+^ or HopX^+^ cardioblasts and to what extent. Also, recent studies^27^,^28^ demonstrated a method for long-term expansion of cardiovascular progenitor cells (CPCs) under chemically defined condition. However, differentiation efficiency of expanded CPCs into cardiomyocytes was low, this did not occur in non-specific differentiation conditions containing only serum. We tried to expand PCBs with the same small molecules used for CPC expansion^27^,^28^. Nevertheless, PCBs could not be expanded keeping cardioblast potential. Moreover, when PCB was cultured as a single cell for clonal assay, the cell immediately arrested and stopped growing or differentiating, implying that cell-to-cell contact is critical for the expansion and maintenance of characters of PCBs. Consequentially, CsAYTE could efficiently induce and expand PCBs but only for a short period, and they lost their proliferative potential after fully differentiating cardiomyocytes. Thus, expansion of PCBs while maintaining their cardioblast potential over a long period remains to be established for applications in cellular therapies for heart failure and studying cardiac specification. The recent elucidation of epigenetic regulation with next-generation sequencing and genome-wide assays for chromatin occupancy by transcription factors has revolutionized our understanding of cardiac specification and differentiation^33^,^34^. Indeed, reports have shown that the transcriptional activation of cardiac-specific enhancers marked by H3K4me1 and, subsequently, H3K4me3 are necessary for cardiac lineage commitment^34^. Consistently, our results showed that activation of chromatin by H3K4me1 and H3K4me3 at the promoters of *meis1* and *tbx5* contributes to cardioblast commitment from Flk1^+^ MPCs.

The characteristics remain to be determined that truly define differentiated cells as functionally implantable cardiomyocytes. Recently, functional attributes such as firing action potentials or oscillating calcium and the results of global transcriptome analysis have been added to the hallmarks of induced cardiomyocytes^29^. In this respect, we defined PCBs as proliferating CLCs that are still in an intermediate state between MPCs and cardiomyocytes using genome-wide, morphologic, and functional analyses. Specifically, PCBs showed less developed mitochondria and cardiac sarcomere structures and showed no electrophysiological activities. In addition, previously reported cardiac progenitors, such as Nkx2.5^+^ reporter cells, were most abundant at days 8–10 after PSC differentiation induction^35^,^36^. In this study, PCBs partly expressed Nkx2.5 and appeared at days 5–6, which is a significantly earlier stage than that for previously known cardiac progenitors. Therefore, we define and characterize PCBs as a unique cardiac lineage cell population, which differs in differentiation stages and immune-phenotypes from previously known cardiac progenitors.

In summary, we uncovered a novel class of cardiac lineage cells, PDGFRα^+^Flk1^−^ cardioblasts from mouse and human PSCs.

## Materials and Methods

Detailed procedures can be found in Supplemental Information.

### PSC and OP9 cell culture

EMG7 mouse ESC, which have an αMHC promoter-driven enhanced GFP gene and E14Tg2a ESC were cultured with LIF incubation. Human iPSC were generated from human foreskin fibroblasts (CRL-2097™, ATCC, Manassas, VA) and cultured on MMC (AG scientific)-treated mouse embryonic fibroblast feeder layers. OP9 cells were used as feeder cell layers for cardiac induction.

### Induction of mouse PSC-derived MPC, cardioblasts and cardiomyocytes

For the induction of Flk1^+^ MPC, ESC and iPSC were cultured without LIF and plated on a 0.1% gelatin-coated dish in differentiation medium for 4.5 days. For induction of cardioblasts and cardiomyocytes, sorted Flk1^+^ MPC were plated onto the MMC-treated confluent OP9 cells or 0.1% gelatin-coated dish.

## Additional Information

**Accession Code:** The Gene Expression Omnibus accession number for gene expression microarray analysis reported in this paper is GSE65791.

**How to cite this article**: Hong, S. P. *et al*. Generation of PDGFRα^+^ Cardioblasts from Pluripotent Stem Cells. *Sci. Rep.* **7**, 41840; doi: 10.1038/srep41840 (2017).

**Publisher's note:** Springer Nature remains neutral with regard to jurisdictional claims in published maps and institutional affiliations.

## Supplementary Material

Supplementary Movie S1

Supplementary Movie S2

Supplementary Information
