# Supplementary material for: Generation of PDGFRα+ Cardioblasts from Pluripotent Stem Cells
Source: Sci Rep. 2017 Feb 6;7:41840. doi: 10.1038/srep41840 (PMC5292955; doi:10.1038/srep41840)
Supplement: Supplementary Information [file srep41840-s3.pdf]

SUPPLEMENTARY INFORMATION

Generation of PDGFRα^+ Cardioblasts from Pluripotent Stem Cells

Seon Pyo Hong, Sukhyun Song, Sung Woo Cho, Seungjoo Lee,
Bong Ihn Koh, Hosung Bae, Kyun Hoo Kim, Jin-Sung Park, Hyo-Sang Do,
Ilkyun Im, Hye Jin Heo, Tae Hee Ko, Jae-Hyeong Park, Jae Boum Youm,
Seong-Jin Kim, Injune Kim, Jin Han, Yong-Mahn Han, Gou Young Koh

Contents:

1. Supplementary Figures S1 – S9 and Their Legends
2. Legends for Movie S1 and S2
3. Supplementary Tables S1
4. Supplementary Materials and Methods

Figure S1

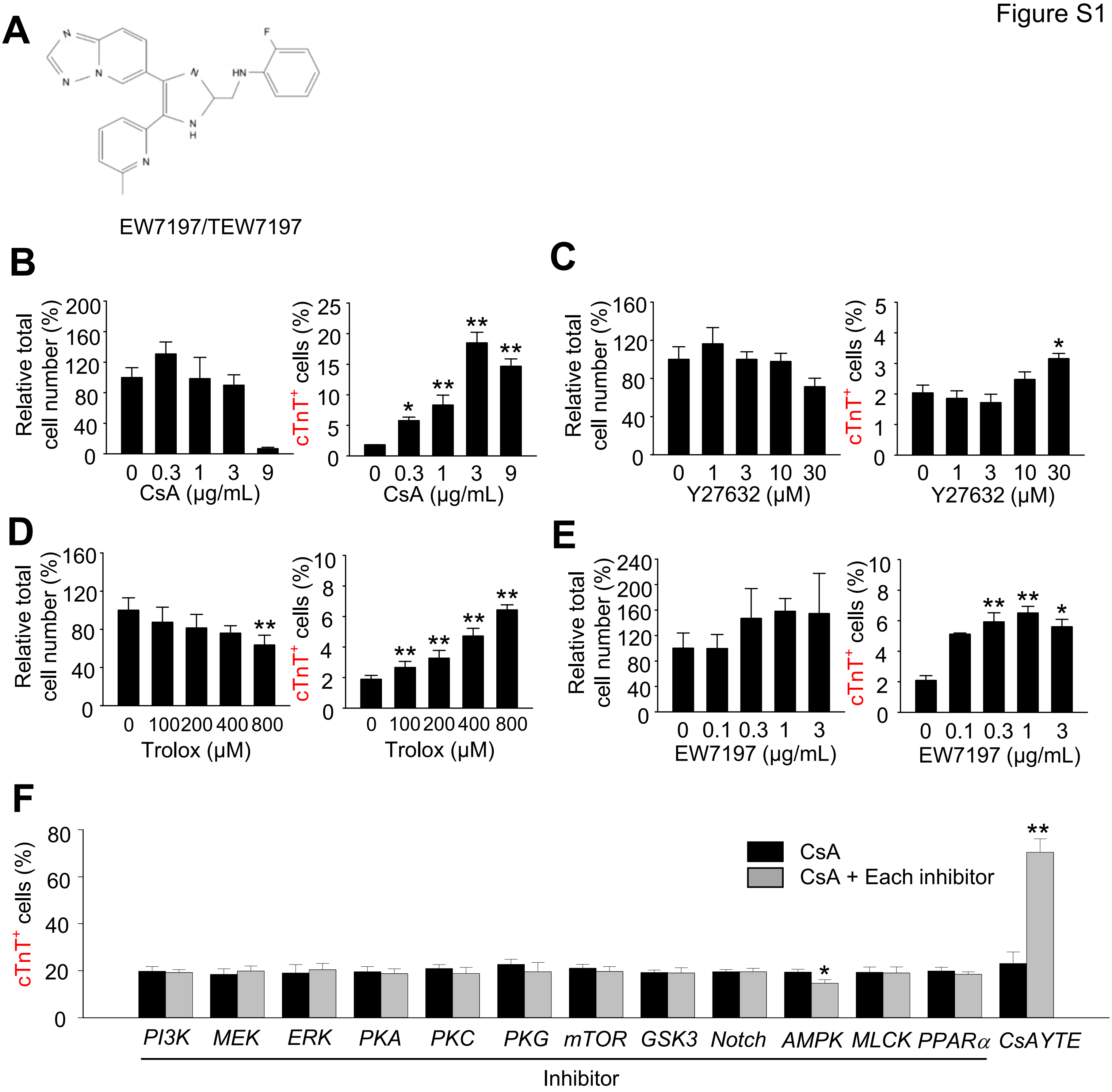

Figure S1. Dose optimization of each reagent and effect of various signal modulators in combination with CsA on cardiac lineage induction.

(A) Chemical structure of EW7197/TEW7197.
(B-F) Mouse ESC-derived Flk1⁺ MPCs were incubated with indicated concentrations of CsA, Y27632, Trolox, EW7197, each inhibitor or CsAYTE. $cTnT^+$ cells were quantified by FACS at day 10.5. Bars indicate relative total cell numbers or percentages of $cTnT^+$ cells. The cell number treated with respective vehicle (0) was regarded as 100%. Each group, n = 3 - 4. *p < 0.05 and **p < 0.01 versus 0 or CsA only. [PI3K inhibitor, LY294002 (20 μM); MEK inhibitor, PD98059 (50 μM), PKA inhibitor, KT5720 (3 μM); PKC inhibitor, Go6976 (0.1 μM); PKG inhibitor, KT5823 (3 μM); mTOR inhibitor, rapamycin (0.02 μM); GSK3 inhibitor, BIO (5 μM); Notch inhibitor, DAPT (10 μM); AMPK inhibitor, Compound C (5 μM); MLC kinase inhibitor, ML-7 (5 μM); PPARα inhibitor, GW6471 (10 μM)]

Figure S2

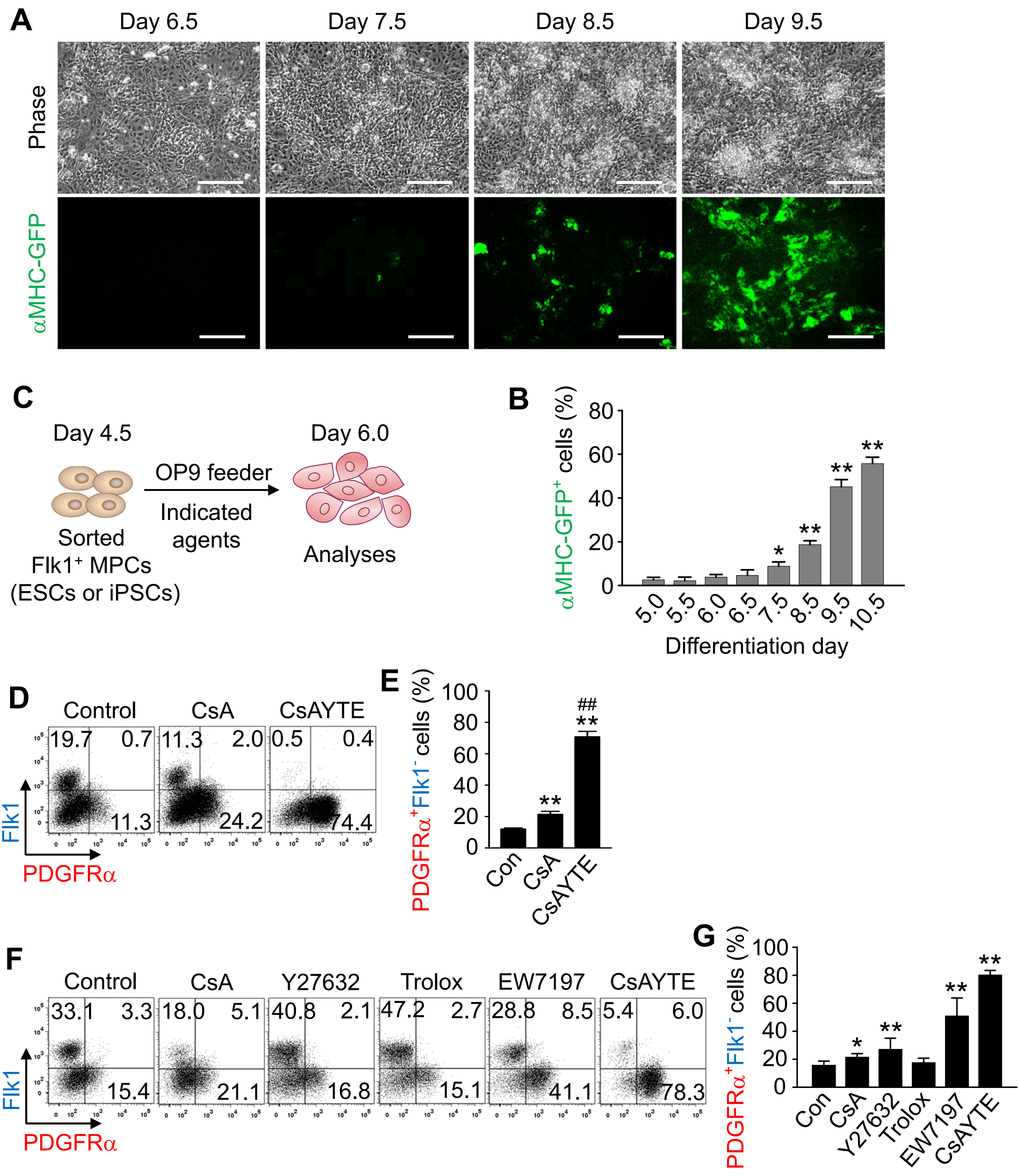

A
Day 6.5
Day 7.5
Day 8.5
Day 9.5
Phase
αMHC-GFP
C
Day 4.5
Sorted
Flk1+ MPCs
(ESCs or iPSCs)
OP9 feeder
Indicated
agents
Day 6.0
Analyses
B
αMHC-GFP+ cells (%)
Differentiation day
5.0
5.5
6.0
6.5
7.5
8.5
9.5
10.5
D
Control
CsA
CsAYTE
19.7
0.7
11.3
11.3
2.0
24.2
0.5
0.4
74.4
Flk1
PDGFRα
E
PDGFRα+Flk1- cells (%)
Con
CsA
CsAYTE
##
F
Control
CsA
Y27632
Trolox
EW7197
CsAYTE
33.1
3.3
15.4
18.0
5.1
21.1
40.8
2.1
16.8
47.2
2.7
15.1
28.8
8.5
41.1
5.4
6.0
78.3
G

Figure S2

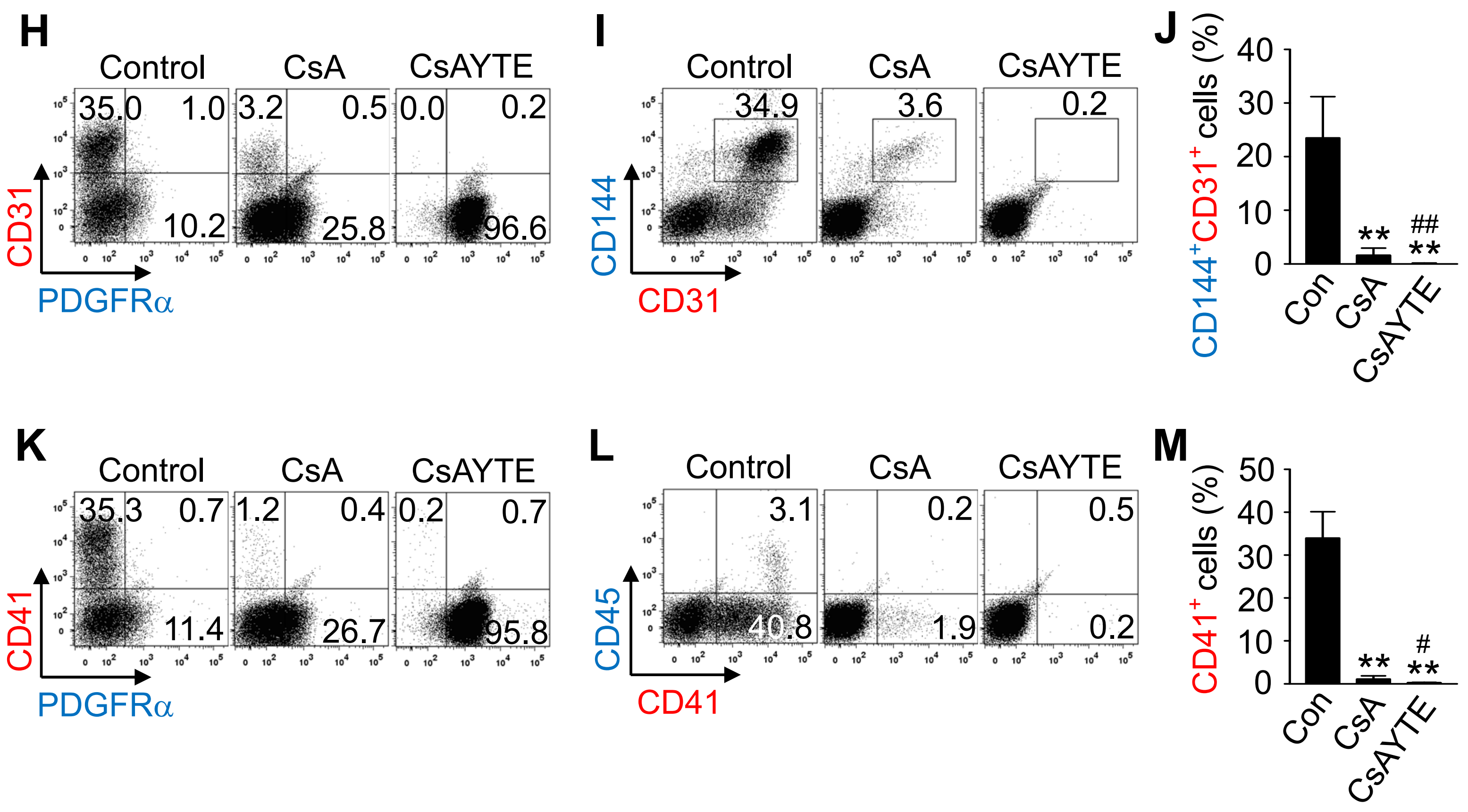

Figure S2. CsAYTE highly and selectively generates PCBs.

(A) Representative phase-contrast and fluorescence images at each differentiation day. Scale bars, 100 μm.
(B) FACS analysis of the percentage of αMHC-GFP$^+$ cells. Each group, n = 3 - 5. *$p < 0.05$ and **$p < 0.01$ versus day 5.0.
(C) Protocol for analyses of PCBs generated from mouse ESCs- or iPSCs-derived Flk1$^+$MPC under incubation with indicated agents in OP9 feeder co-culture system.
(D and E) Representative FACS analysis and the percentage of mouse iPSC-derived PDGFRα$^+$Flk1$^-$ cells at day 6.0 incubated with control vehicle (Con), CsA, and CsAYTE. Each group, n = 4. **$p < 0.01$ versus Con; ##$p < 0.01$ versus CsA.
(F and G) Representative FACS analysis and the percentage of PDGFRα$^+$Flk1$^-$ cells at day 6.0 incubated with the indicated agents. Each group, n = 3 - 4. **$p < 0.01$ versus Con.
(H-J) Representative FACS analysis of PDGFRα and CD31 expression and representative FACS analysis and the percentage of CD144$^+$CD31$^+$ endothelial cells at day 6.0 incubated with Con, CsA (3 μg/mL) and CsAYTE. Each group, n = 4. **$p < 0.01$ versus Con; ##$p < 0.01$ versus CsA.
(K-M) Representative FACS analysis of PDGFRα and CD41 expression and representative FACS analysis and the percentage of CD41$^+$ early hematopoietic cells at day 6.0 incubated with Con, CsA (3 μg/mL) and CsAYTE. Each group, n = 4. **$p < 0.01$ versus Con; #$p < 0.05$ versus CsA.

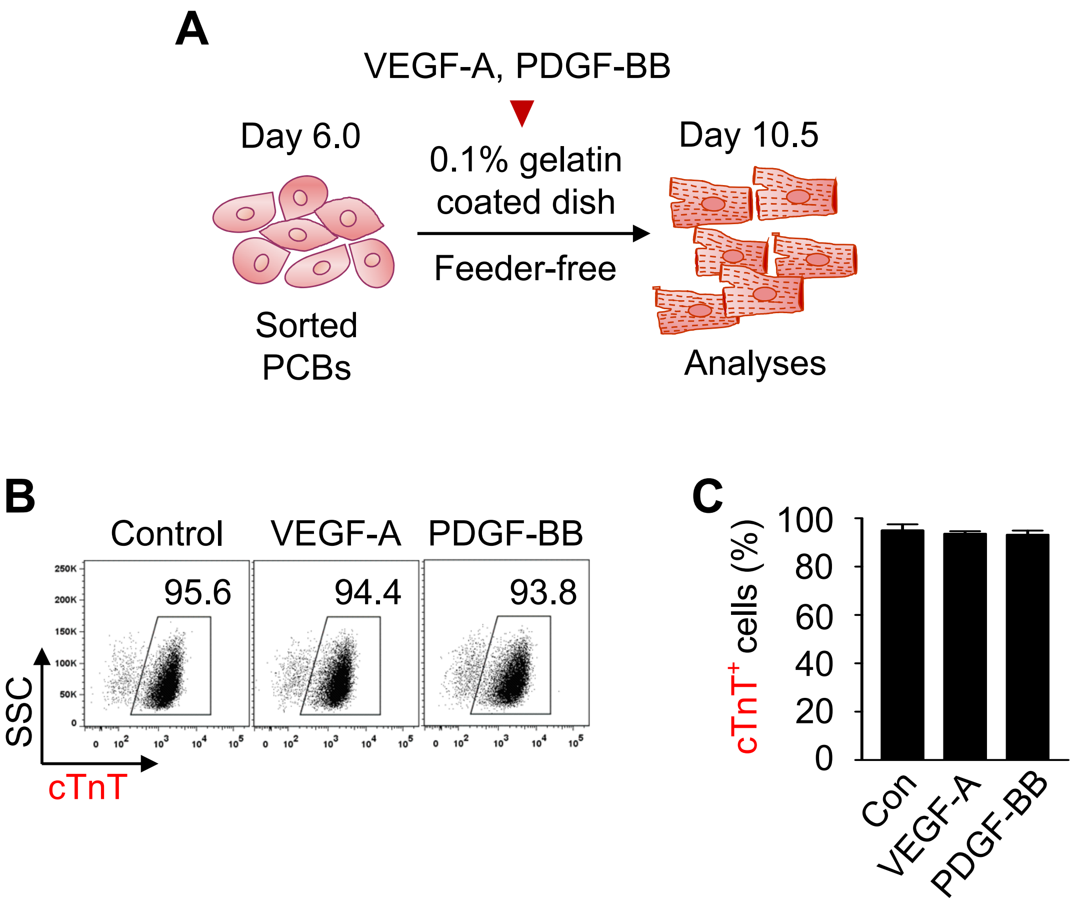

Figure S3. Differentiation potential of PCBs is limited into cardiomyocytes even under non-cardiac specific condition.

(A) Protocol for analyses of PCB-derived cardiomyocyte differentiation in non cardiomyocyte-specific condition.
(B and C) Representative FACS analysis and percentages of $cTnT^+$ cells at day 10.5 in the presence of VEGF-A (200 ng/ml) and PDGF-BB (50 ng/ml). Each group, n = 4.

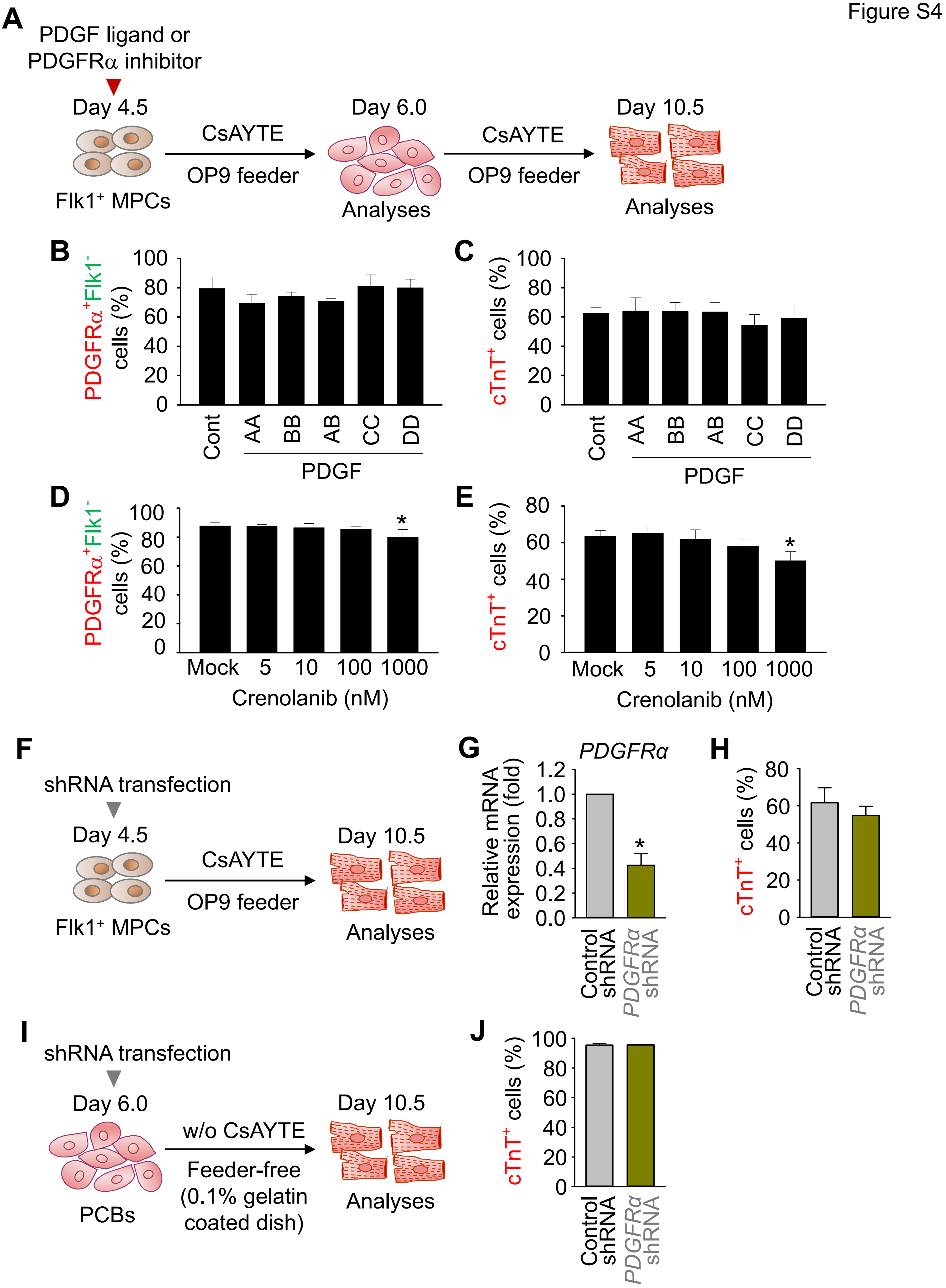
A
PDGF ligand or
PDGFRα inhibitor
Day 4.5
CsAYTE
OP9 feeder
Flk1+ MPCs
Day 6.0
Analyses
CsAYTE
OP9 feeder
Day 10.5
Analyses
B
PDGFRα+Flk1- cells (%)
100
80
60
40
20
0
Cont
AA
BB
AB
CC
DD
PDGF
C
cTnT+ cells (%)
100
80
60
40
20
0
Cont
AA
BB
AB
CC
DD
PDGF
D
PDGFRα+Flk1- cells (%)
*
Mock
5
10
100
1000
Crenolanib (nM)
E
cTnT+ cells (%)
80
60
40
20
0
*
Mock
5
10
100
1000
Crenolanib (nM)
F
shRNA transfection
Day 4.5
CsAYTE
OP9 feeder
Flk1+ MPCs
Day 10.5
Analyses
G
PDGFRα
Relative mRNA expression (fold)
1.2
1.0
0.8
0.6
0.4
0.2
0.0
*
Control shRNA
PDGFRα shRNA
H
cTnT+ cells (%)
80
60
40
20
0
Control shRNA
PDGFRα shRNA
I
shRNA transfection
Day 6.0
w/o CsAYTE
Feeder-free
(0.1% gelatin
coated dish)
PCBs
Day 10.5
Analyses
J
cTnT+ cells (%)
100
80
60
40
20
0
Control shRNA
PDGFRα shRNA

Figure S4. PDGFRα is a simple surface marker of PCBs, rather than a functional receptor during cardiac differentiation.

(A) Protocol for analyses of PCBs generation and their cardiac differentiation from $Flk1^+$ MPCs.
(B and C) Percentages of $PDGFR\alpha^+Flk1^-$ cells at day 6.0, and $cTnT^+$ cells at day 10.5 in the presence of each PDGF ligand (50 ng/ml), PDGF-AA, -BB, -AB, -CC and -DD. Each group, n = 3 - 4.
(D and E) Percentages of $PDGFR\alpha^+Flk1^-$ cells at day 6.0, and $cTnT^+$ cells at day 10.5 in the indicated concentration of PDGFRα inhibitor crenolanib. Each group, n = 3 - 4.
(F) Protocol for analyses of cardiac differentiation under transfection of PDGFRα shRNA to $Flk1^+$ MPCs at day 4.5
(G) Relative expression PDGFRα mRNA at day 6.0 after the transfection of PDGFRα shRNA. n = 4. *$p < 0.05$ versus control shRNA.
(H) Percentage of $cTnT^+$ cells after the transfection of PDGFRα shRNA to $Flk1^+$ MPCs. Each group, n = 4. *$p < 0.05$ versus control shRNA.
(I) Protocol for analyses of cardiac differentiation after the transfection of PDGFRα shRNA to FACS-sorted PCBs.
(J) Percentage of $cTnT^+$ cells after the transfection of PDGFRα shRNA to FACS-sorted PCBs. Each group, n = 4. *$p < 0.05$ versus control shRNA.

Figure S5

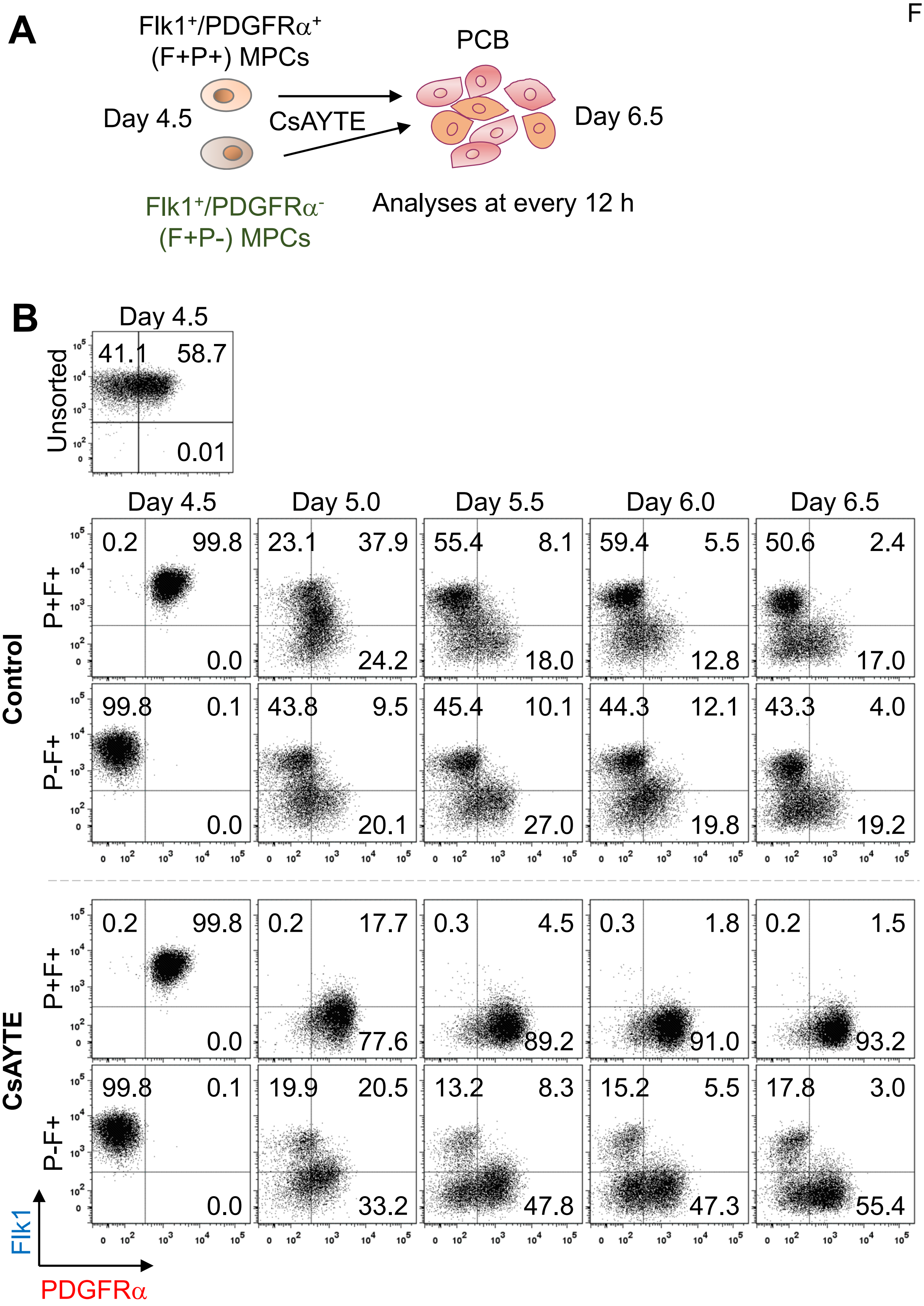

Figure S5. Rapid conversion of Flk1⁺ MPCs into PCBs by CsAYTE.

(A) Protocol for the analyses of phenotype changes. The Flk1⁺ MPCs isolated at day 4.5 were divided into Flk1⁺PDGFRα⁺ (F+P+) MPCs and Flk1⁺PDGFRα⁻(P-F+) MPCs, incubated with or without CsAYTE over 2 days, and examined the phenotypes at every 12 h.
(B) Representative FACS analysis for Flk1 and PDGFRα.

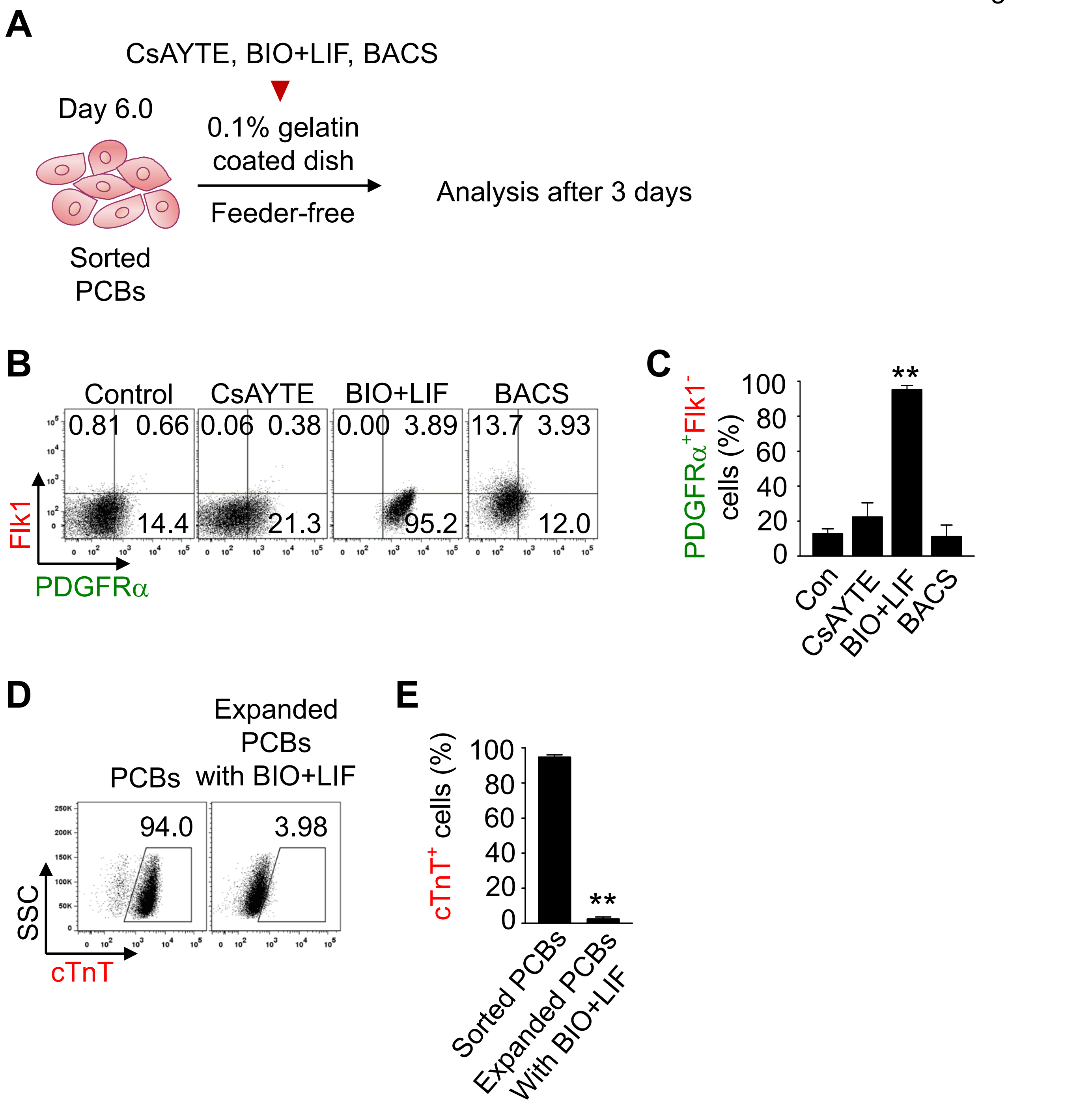

Figure S6. PCBs can be just efficiently induced and expanded by CsAYTE within short period rather than expanded keeping their cardiogenic potential for long-term.

(A) Protocol for analyses of PCB expansion under incubation with control vehicle, CsAYTE, BIO (2.5 μM)+LIF(10^3 units/ml), and BACS (5 ng/ml BMP4, 10 ng/ml Activin A, 3 μM CHIR99021, and 2 μM SU5402).
(B and C) Representative FACS analysis and the percentage of PDGFRα$^+$Flk1$^-$ cells 3 days after seeding PCBs purified on day 6.0 incubated with control vehicle (Con), CsAYTE, BIO (2.5 μM)+LIF(10^3 units/ml), and BACS (5 ng/ml BMP4, 10 ng/ml Activin A, 3 μM CHIR99021, and 2 μM SU5402). Each group, n=4. **$p < 0.01$ versus Con.
(D and E) Representative FACS analysis and the percentages of cTnT$^+$ cells to confirm the cardiac differentiation capacity. Expanded PCBs with BIO+LIF were differentiated without BIO+LIF for 5 days, and cTnT$^+$ cells were analyzed. Each group, n = 4. **$p < 0.01$ versus Sorted PCBs.

Figure S7

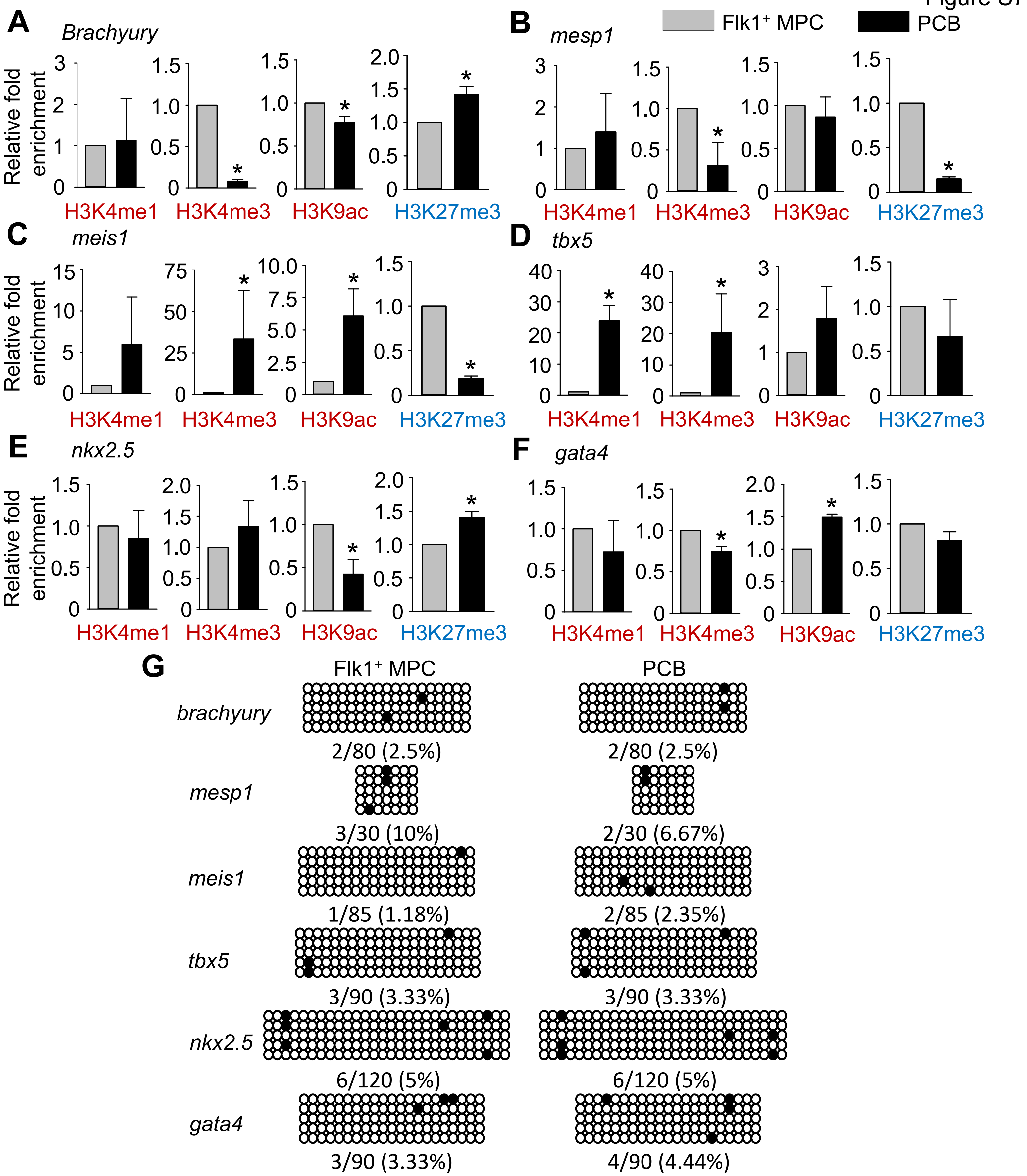

Figure S7. Histone modification during PCBs generation.

(A-F) ChIP enrichments of *brachyury, mesp1, meis1, tbx5, nkx2.5,* and *gata4*, which are marked by H3K4me1, H3K4me3, H3K9ac, and H3K27me3 at their promoters in Flk1^{+} MPCs and PCBs. Each group, n = 3. *$p < 0.05$ versus Flk1^{+} MPC.
(G) DNA methylation status of *brachyury, mesp1, meis1, tbx5, nkx2.5* and *gata4* at their promoters in Flk1^{+} MPCs and PCBs.

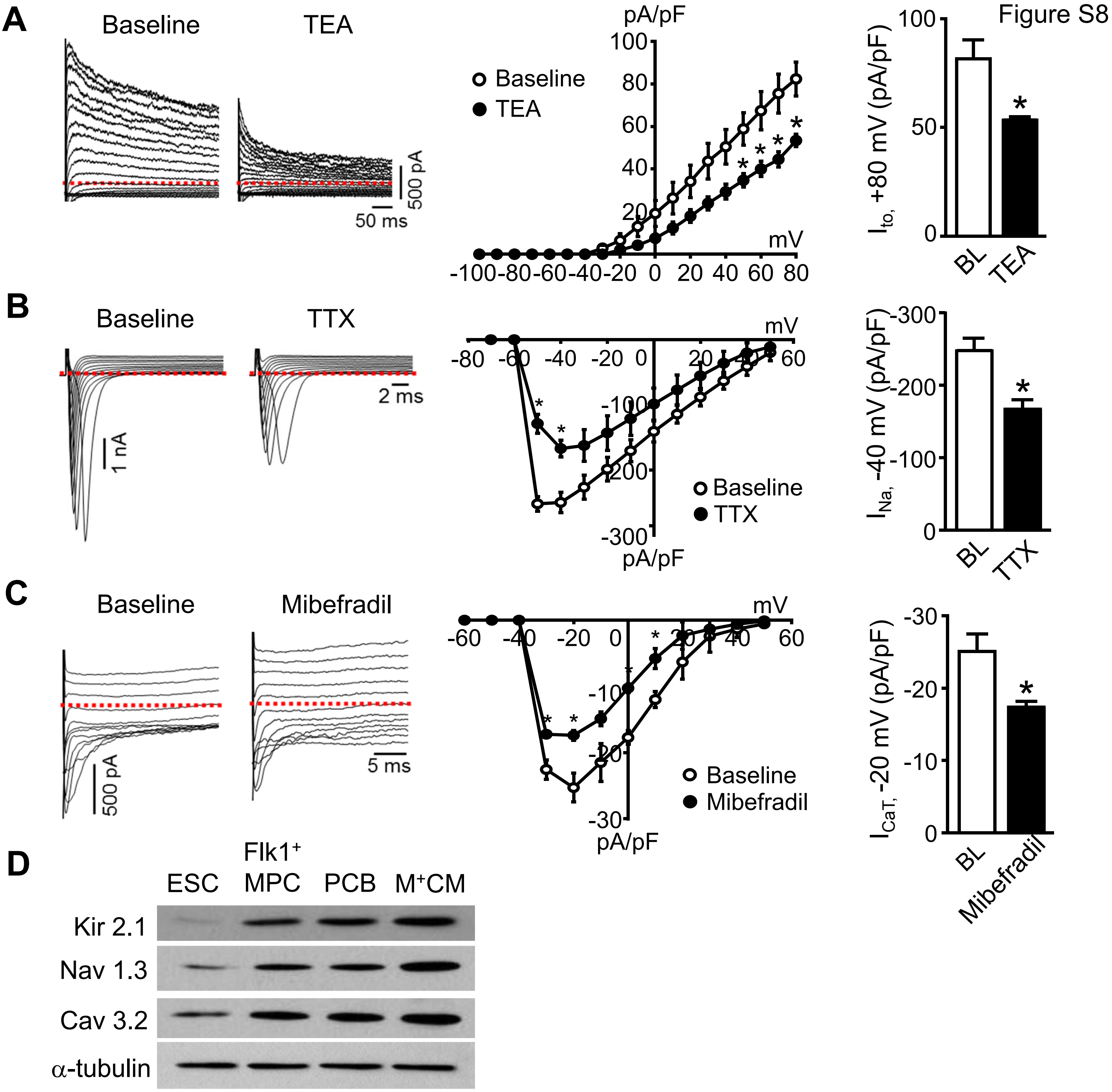

Figure S8. Electrophysiological characterizations of PCBs.

(A) Delayed rectifying K^+ current (I_K) evoked by depolarizing test pulses between -100 and +80 mV in 10 mV increment from a holding potential of -80 mV at 5 s interval. I_K is effectively inhibited by TEA at test potentials between +50 and +80 mV. Each group, n = 3. *$p < 0.05$ versus baseline.
(B) Na^+ current (I_{Na}) evoked by depolarizing test pulses between -70 mV and +50 mV in 10 mV increment from a holding potential of -80 mV at 5 s interval. I_{Na} is effectively inhibited by TTX at test potentials between -50 and -40 mV. Each group, n = 3. *$p < 0.05$ versus baseline.
(C) T-type Ca^{2+} current (I_{CaT}) activated by depolarizing test pulses between -60 and +50 mV in 10 mV increment from a holding potential of -40 mV at 5 s interval. I_{CaT} is effectively inhibited by mibefradil at test potentials between -30 and +10 mV. Each group, n = 3. *$p < 0.05$ versus baseline.
(D) Western blot analysis for expressions of ion channel proteins in the indicated cells.

Figure S9

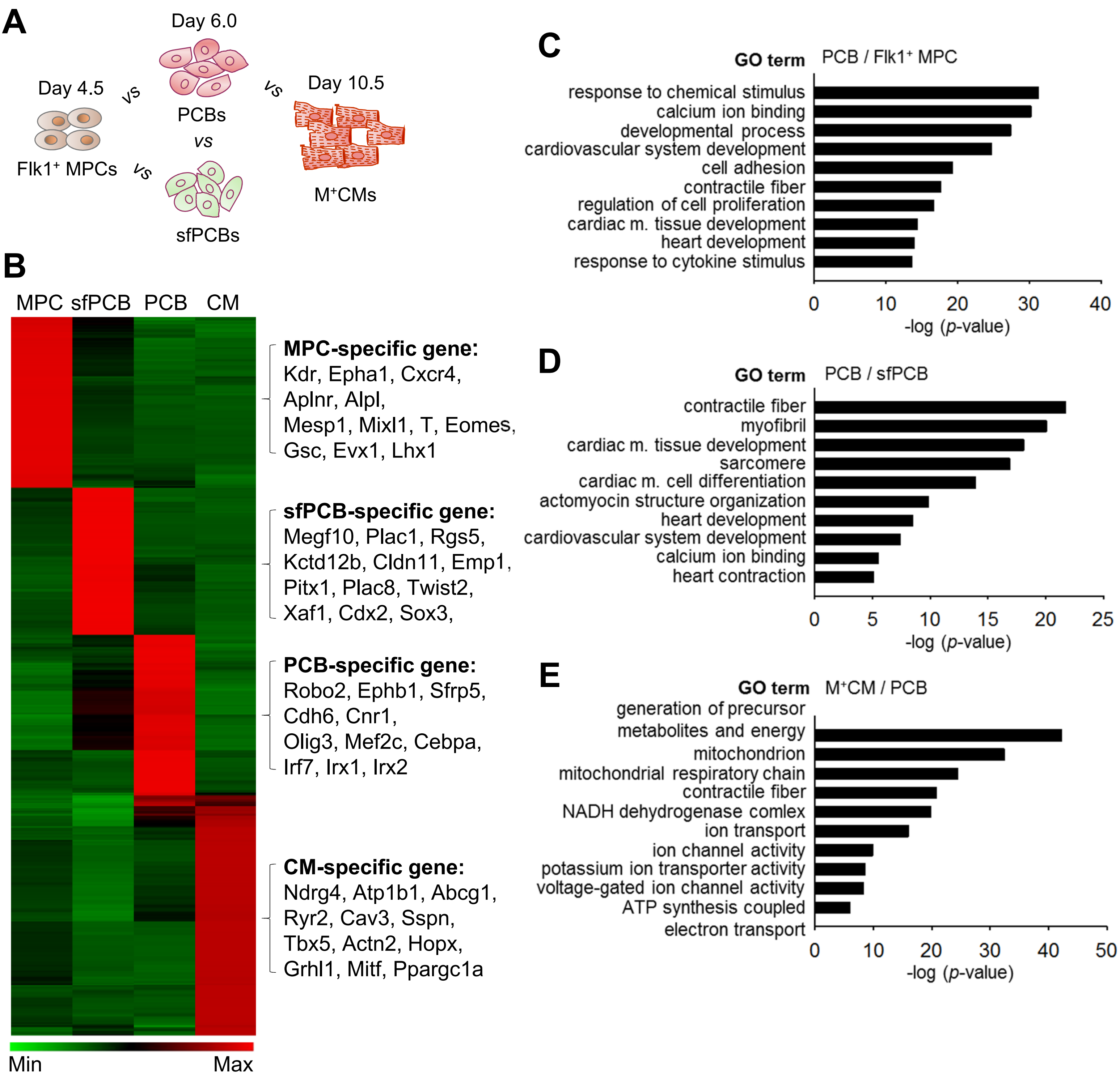

Figure S9. Global transcriptome analysis identifies PCBs as immature CLCs.

(A) Comparison and sampling for microarray analysis of Flk1$^+$ MPCs, PCBs, spontaneously formed PCBs (sfPCBs) which were induced without CsAYTE incubation, and M$^+$CMs.
(B) Microarray gene expression heat map and representative gene lists of plasma membrane proteins and transcriptional regulators for each stage. Red and green represent up- and down-regulations, respectively.
(C) Gene ontology analysis comparing PCBs and Flk1$^+$ MPCs.
(D) Gene ontology analysis comparing PCBs and sfPCBs.
(E) Gene ontology analysis of comparing M$^+$CMs and PCBs.

MOVIE LEGENDS

Movie S1. Beating cardiomyocyte colonies incubated with CsAYTE in OP9 co-culture system at day 10.5.

Movie S2. Tracing of Flk1^{+} MPCs differentiation process incubated with CsAYTE.

Table S1. Primers for real time PCR.

Mouse *oct4*	Forward	5'- TCTTTCCACCAGGCCCCCGGCTC -3'
	Reverse	5'- TGCGGGCGGACATGGGGAGATCC -3'
Mouse *nanog*	Forward	5'- AGGGTCTGCTACTGAGATGCTCTG -3'
	Reverse	5'- CAACCACTGGTTTTTCTGCCACCG -3'
Mouse *sox2*	Forward	5'- TAGAGCTAGACTCCGGGCGATGA -3'
	Reverse	5'- TTGCCTTAAACAAGACCACGAAA -3'
Mouse *brachyury*	Forward	5'- CTATGCTCATCGGAACAGCTCTCCA -3'
	Reverse	5'- CTCACAGACCAGAGACTGGGATAC -3'
Mouse *mesp1*	Forward	5'- CCATCGTTCCTGTACGCAGAAACAG -3'
	Reverse	5'- AGACAGGGTGACAATCATCCGTTGC -3'
Mouse *meis1*	Forward	5'- CATCCACTCGTTCAGGAGGAACC -3'
	Reverse	5'- CTACACTGTTGTCCAAGCCATCAC -3'
Mouse *tbx5*	Forward	5'- CGCCTCTGGAGCCTGATTCCAAAG -3'
	Reverse	5'- GTGCCCACTTCGTGGAACTTCAGC -3'
Mouse *nkx2.5*	Forward	5'- CACGCCTTTCTCAGTCAAAGACATCC -3'
	Reverse	5'- CTGGGAAAGCAGGAGAGCACTTGG -3'
Mouse *isl1*	Forward	5'- AGACCCTCTCAGTCCCTTGCATC -3'
	Reverse	5'- CATCTCCACTAGTTGCTCCTTCATG -3'
Mouse *gata4*	Forward	5'- CCCTCTTTGTCATTCTTCGCTGGAG -3'
	Reverse	5'- GATTTGCGGTTGCTCCAGAAATCGTG -3'
Mouse *hand2*	Forward	5'- CAAGATCAAGACACTGCGCCTGG -3'
	Reverse	5'- TCGTTGCTGCTCACTGTGCTTTTC -3'
Mouse *tnnt2*	Forward	5'- GACCTGTGTGCAGTCCCTGTTCAG -3'
	Reverse	5'- CTTGCTCGTCCTCCTCTTCTTCAC -3'
Mouse *myl7*	Forward	5'- ATCAGACCTGAAGGAGACCTATTCC -3'
	Reverse	5'- AAGGCACTCAGGATGGCTTCCTC -3'
Mouse *pgc1α*	Forward	5'- GCGCCGTGTGATTTACGTT -3'
	Reverse	5'- AAAACTTCAAAGCGGTCTCTCAA -3'
Mouse *connexin43*	Forward	5'- GTGTCTGTGCCCACACTCCTGTAC -3'
	Reverse	5'- CTCAGCAGGCCACCTCTCATCTTC -3'
Mouse *pdgfrα*	Forward	5'- TTTCTGGTCCTCAGCTGTCTCCTC -3'
	Reverse	5'- TTCACTCTCCCCAACGCATCTCAG -3'
Mouse *beta actin*	Forward	5'- GCTCTTTTCCAGCCTTCCTT -3'
	Reverse	5'- CTTCTGCATCCTGTCAGCAA -3'

SUPPLEMENTARY MATERIALS AND METHODS

Mouse PSCs and OP9 cell culture

EMG7 mouse ESCs, which have an αMHC promoter-driven enhanced GFP gene, E14Tg2a ESCs, and OP9 cells were generated as described previously[1-3] and transferred to KAIST. Mouse iPSCs derived from FVB strain, which were generated as described previously[4], were a generous gift from Drs. Hyun-Jai Cho and Hyo-Soo Kim (Seoul National University Hospital).

Human iPSC culture

Human iPSCs were generated from human foreskin fibroblasts (CRL-2097, ATCC) by ectopically expressing 4 transcription factors OCT4, SOX2, KLF4, and c-MYC as previously described[5]. Human iPSCs were maintained on MMC-treated mouse embryonic fibroblast feeder layers in Dulbecco's modified Eagle medium (DMEM)/F-12 (Invitrogen) supplemented with 20% Knockout Serum Replacement (Invitrogen), 1% non-essential amino acids (Invitrogen), 1% penicillin–streptomycin (Invitrogen), 0.1 mM 2-mercaptoethanol (Sigma–Aldrich), and 4 ng/ml basic fibroblast growth factor (bFGF; R&D Systems). The medium was changed daily.

Induction of mouse PSC-derived MPCs, cardioblasts and cardiomyocytes

For the induction of Flk1$^+$ MPCs, ESCs and iPSCs were cultured without leukemia inhibitory factor (LIF, Millipore) and plated on a 0.1% gelatin-coated dish at a cell density between 1×10^3 and 1.5×10^3 cells cm^{-2} in the differentiation medium, which is αMEM (Invitrogen) containing 10% fetal bovine serum (FBS, Welgene), 0.1 mM of 2-mercaptoethanol (Invitrogen), 2 mM of L-glutamine (Invitrogen) and 50 U/ml of penicillin-streptomycin (Invitrogen). Medium was changed every other day for 4.5 days. At day 4.5, differentiated ESC and iPSC were harvested with 0.25% trypsin-EDTA (Invitrogen), and antigen recovery was performed in the differentiation medium for 30 min in an incubator. Then, cells were washed using 2% FBS in phosphate buffered saline (PBS) and incubated with biotin-conjugated anti–mouse Flk1 antibody (clone AVAS12a1, eBioscience) and anti-streptavidin MicroBeads (Miltenyi Biotec). Flk1$^+$ MPCs were sorted by AutoMACS Pro Separator (Miltenyi Biotec). For induction of cardiac specification, sorted Flk1$^+$ MPCs were plated onto the mitomycin C (AG Scientific)-treated confluent OP9 cells at a density of 5-10 × 10^3 cells cm^{-2} in the medium containing 3 μg/mL of CsA, 10 μM of Y27632, 400 μM of Trolox, and 1 μg/mL of EW7197 (CsAYTE). The medium was refreshed every other day. For the induction

of cardiac specification in a feeder-free system, $Flk1^+$ MPCs were plated on a 0.1% gelatin coated dish at cell density 1 × 10^4 cells cm^{-2} without OP9 cells in the medium containing CsAYTE and 2 μM of IWR1. The medium was refreshed every other day. For differentiation of PCBs into cardiomyocytes in a feeder-free system, PCBs were sorted by FACS and plated on a 0.1% gelatin-coated dish at cell density 6 × 10^5 cells cm^{-2} without OP9 cells. Cells were cultured in the differentiation medium which was changed every other day. To validate whether PCBs can be expanded, purified PCBs at day 6.0, were seeded onto a 0.1% gelatin-coated culture plate at a low-density (6.5 × 10^4 cells/cm^2), and incubated them with either control vehicle, CsAYTE, BIO (2.5 μM) + LIF (10^3 units/ml), or BACS (5 ng/ml BMP4, 10 ng/ml Activin A, 3 μM CHIR99021, and 2 μM SU5402).

Induction of human iPSC-derived cardioblasts and cardiomyocytes

Human iPSC-derived cardiomyocyte differentiation was induced as previously reported[6]. For cardiac lineage induction, human iPSCs were plated onto Matrigel-coated dishes at a density of 1.0 × 10^5 cells cm^{-2} and cultured in mTeSR1 supplemented with bFGF (4 ng/ml) for 2–3 days. Then, culture medium was replaced with RPMI+B27 medium (RPMI1640, 2 mM L-glutamine, and 1 x B27 supplement without insulin) supplemented with activin A (50 ng/ml, R&D Systems), human BMP4 (25 ng/ml, R&D Systems) and CHIR99021 (3 μM, Cayman Chemical) for 2 days. The culture medium was subsequently changed with RPMI+B27 supplemented with CsAYTE, 100 ng/ml of dickkopf-related protein 1 (Dkk1; R&D Systems) and 25 ng/ml of vascular endothelial growth factor-A (VEGF; R&D Systems) for 2 days. At day 4, the culture medium was replaced with RPMI+B27 without growth factors. The medium was changed every 1–2 days. Beating cardiomyocytes were observed at day 8–9.

Flow cytometry analysis and cell sorting

The cells were harvested with 0.25% trypsin-EDTA or dissociation buffer (Invitrogen). To analyze live cells, antigen recovery was performed in the differentiation medium for 30 min in an incubator and the cells were incubated for 20 min with the following antibodies: phycoerythrin (PE)-conjugated anti–mouse PDGFRα (eBioscience, 12-1401, clone APA5, 1:100), allophycocyanin (APC)-conjugated anti–mouse Flk1 (BioLegend, 136406, clone AVAS12a1, 1:50), PE-conjugated anti-mouse CD31 (eBioscience, 12-0311, clone 390, 1:100), APC-conjugated anti–mouse CD144 (eBioscience, 17-1441, clone BV13, 1:100), PE-conjugated anti-mouse CD41 (BD Biosciences, 558040, clone MWReg30, 1:100), APC-conjugated anti-mouse CD45 (eBioscience, 17-0451, clone 30-F11, 1:100) antibodies, PE-

conjugated anti-human KDR (R&D Systems, FAB357P, clone 89106, 1:50), and APC-conjugated anti-human PDGFRα (R&D Systems, FAB1264A, clone PRa292, 1:50). In live cell analysis and sorting, dead cells were excluded using 4,6-diamidino-2-phenylindole (DAPI, Sigma, D8417, 1:1000), and OP9 cells were excluded from Flk1^{+} MPC by gating in flow cytometry. The differentiated cardiomyocytes were sorted using αMHC-GFP. To analyze cTnT^{+} cells, the cells were permeabilized with Cytofix/Cytoperm solution (BD Biosciences) for 15 min. After permeabilization, the cells were incubated for 30 min with anti-mouse cTnT (Thermo Scientific, MA5-12960, Clone 13-11, 1:1000) monoclonal antibody. After washing in 10% Perm/Wash buffer (BD Biosciences), the cells were incubated for 10 min with Cy5-conjugated anti–mouse IgG antibody (Invitrogen, A10524, 1:1000). The cells were washed with 10% Perm/Wash buffer (BD Biosciences) and then analyzed. Analyses and sorting were performed by FACS Aria II (Beckton Dickinson). Data were analyzed using FlowJo Version 7.5.4 software (TreeStar).

Immunofluorescence staining and visualization of cells

The cells were fixed with 4% paraformaldehyde (PFA) and blocked with 5% goat (or donkey) serum in PBST (0.1% Tween 20 in PBS) for 1 h at room temperature (RT). The cells were stored overnight at 4°C with the following primary antibodies: anti-mouse cTnT (Thermo Scientific, MA5-12960, Clone 13-11, 1:500) and anti-mouse α-actinin (Sigma Aldrich, A7811, clone EA-53, 1:100) monoclonal antibody, anti–mouse PDGFRα (eBioscience, 16-1401, clone APA5, 1:200), anti–mouse Flk1 (Cell Signaling, 2479, clone 55B11, 1:200), and anti-moues Nkx 2.5 polyclonal antibody (Santa Cruz Biotechnology, sc-14033, H-114, 1:200). After being washed with PBST 3 times, the cells were incubated for 2 h at RT with the following secondary antibodies: Cy5-conjugated anti–mouse IgG antibody (Invitrogen, A10524, 1:1000), FITC-conjugated anti-rat IgG (Jackson Immuno Research, 112-095-003, 1:1000), and Alexa Fluor 647-conjugated anti-rabbit IgG (Invitrogen, A-21244, 1:1000) antibodies. Nuclei were stained with DAPI (Sigma, D8417, 1:1000). After being stained with the antibodies, the cells were mounted in fluorescent mounting medium (DAKO). Immunofluorescence staining of mitochondria was performed using MitoTracker Orange CMTMRos probe (Invitrogen), with which the cells were incubated for 30 to 60 min at 37°C in serum-free medium before fixation. Immunocytochemistry stained images were obtained using an LSM780 confocal fluorescence microscope (Carl Zeiss). Live images of cardiomyocyte differentiation process and αMHC GFP^{+} cardiomyocytes were obtained using Axiovert 200M microscope (Carl Zeiss) equipped with AxioCam MRm (Carl Zeiss). Images were analyzed using ImageJ software (http://imagej.nih.gov/ij/, 1.47V, NIH, USA). Phase-

contrast images including beating cardiomyocytes were obtained using an Infinity X digital camera and DpxView LE software (DeltaPix).

Assays for cell cycle

To determine the cell cycle, BrdU/7-AAD cell cycle analysis was performed according to the manufacturer's instructions (BD Biosciences). Briefly, cells were incubated with BrdU (1 mM) for 1 h, dissociated with 0.25% trypsin-EDTA, fixed, permeabilized, and fixed once more, followed by 1 h incubation with DNase I (200 U) at 37°C. After incubation with APC-conjugated anti-BrdU antibody for 20 min at RT, the cells were stained with 7-AAD. The cells were analyzed by FACS Aria II and the data were analyzed using FlowJo software.

Quantitative real time PCR

Total RNA was extracted using Trizol RNA extraction kit (Invitrogen) according to the manufacturer's instructions. Total RNA was reverse transcribed into cDNA using GoScript^{TM} cDNA synthesis system (Promega). cDNA was applied for quantitative real-time PCR using FastStart SYBR Green Master mix (Roche) and Bio-rad S1000 Thermocycler with the indicated primers (Supplementary Table 2). Beta-actin was used as a reference gene and the results were presented as relative values to control using the ΔΔCt method.

Short Hairpin RNA (shRNA)-Mediated Silencing of PDGFRα

PDGFRα was silenced in Flk1$^+$ MPCs and PCBs derived from mouse ESC using PDGFRα-shRNA lentiviral transduction particles (SHCLNV-NM_011058, Sigma-Aldrich). Control cells were transduced with non-target shRNA lentiviral particles (SHC216V, Sigma-Aldrich) according to the manufacturer's instruction. Transduction of Flk1$^+$ MPCs or PCBs was carried out by spinoculation (1,000 g) for 90 min at 4°C in the presence of 8 μg/ml polybrene reagent.

Western Blotting

Cells were homogenized in lysis buffer (20mM Tris-HCl, 150mM NaCl, 10mM EDTA, 50mM NaF, and 25mM NaVO4) and centrifuged at 13,000 rpm for 10 min at 4°C. After centrifugation, 30 μg of proteins were subjected to 10% sodium dodecyl sulfate-polyacrylamide gels and transferred onto nitrocellulose membranes. Membranes were incubated with either Cav3.2 (Alomone), Kir2.1 (Alomone), Nav1.3 (Alomone), α-tubulin (Santa cruz), or Actin (Sigma Aldrich) primary antibodies and then with goat anti-rabbit or mouse IgG-HRP (Abcam) secondary antibodies. The immunoreactive protein bands were

detected using SuperSignal West Pico enhanced chemiluminescence system (Thermo Fisher Scientific Inc) and visualized using LAS-3000 PLUS (Fuji Photo Film Company).

Transmission electron microscopic analysis

The cells were fixed in 2.5% glutaraldehyde in PBS at 4°C overnight, and then with 1% osmium tetroxide in PBS for 2 h. The tissues were washed, dehydrated, embedded, and then semi-thin sections were cut (0.5–1 μm). Further ultra-sectioning (60–90 nm) was performed and then the slices were double stained with uranyl acetate and lead citrate, and imaged using a JEM 1200 EX2 electron microscope (Jeol). Developed images were scanned on a flatbed scanner (Umax PowerLook 1100, Fremont) and analyzed using ImageJ software.

Electrophysiology

Action potentials (APs) and ion currents were recorded from cells placed onto the recording chamber of microscope by using Axopatch 200B amplifier (Axon Instrument) at room temperature (23±1°C). Normal Tyrode (NT) solutions were used during seal formation and it contained 143 mM NaCl, 5.4 mM KCl, 0.5 mM $MgCl_2$, 1.8 mM $CaCl_2$, 5.5 mM glucose, and 5 mM N-[2-hydroxyethyl]piperazine-N-[2-ethanesulfonic acid] (HEPES). pH was adjusted to 7.4 with 1 M NaOH. For measurement of APs or K^+ current, we used K^+-rich pipette filling solutions containing 140 mM KCl, 1 mM $MgCl_2$, 5 mM MgATP, 5 mM ethyleneglycol-bis(β-aminoethyl ether)-N,N,N',N'-tetraacetic acid (EGTA), 5 mM glucose, and 5 mM HEPES, titrated to pH 7.2 with 1 M KOH. For measurement of Ca^{2+} or Na^+ currents, the bathing solution was switched from NT to a solution containing 120 mM NaCl, 5 mM KCl, 1 mM $MgCl_2$, 3.6 mM $CaCl_2$, 20 mM TEA, and 10 mM HEPES, titrated to pH 7.4 with 1 M NaOH. Pipette filling solutions contained 140 mM CsCl, 5 mM glucose, 3 mM MgATP, 10 mM EGTA, and 10 mM HEPES, titrated to pH 7.2 with 1 M CsOH. Patch pipettes were pulled from thin-walled borosilicate capillaries (Clark Electromedical Instruments) using a PP-83 vertical puller (Narishige). GΩ seal formation and membrane rupture were achieved by applying negative pressure onto the membrane patch and only whole-cell patches with series resistance < 5 MΩ were selected for recording. All the recordings were carried out at least 5 min after achieving whole-cell configuration to allow cells to be completely dialyzed with pipette-filling solution. Spontaneous APs were recorded in current-clamp mode while ion currents were recorded in voltage-clamp mode. The voltage and current signals were filtered at 10 kHz, 4-pole Bessel type low-pass filter and sampled at a rate of 4 kHz for voltage and 27 kHz for ion current. All experimental parameters, such as pulse generation and data

acquisition, were controlled using our own software (PatchPro). The liquid junction potentials between bathing and pipette filling solution, which were calculated based on ionic mobility, were < 5 mV. TEA, TTX, and mibefradil were used to block delayed rectifying K^+ channels, voltage-gated Na^+ channels, and T-type Ca^{2+} channels, respectively.

Microarray analysis

For control and test RNAs, synthesis of target cRNA probes and hybridization were performed using Agilent's Low RNA Input Linear Amplification kit (Agilent Technology) according to the manufacturer's instructions. Briefly, each 1 μg of total RNA and T7 promoter primer mix were incubated at 65°C for 10 min. cDNA master mix (5X First strand buffer, 0.1M DTT, 10mM dNTP mix, RNase-Out, and MMLV-RT) was prepared and added to the reaction mixer. The samples were incubated at 40°C for 2 h and then the reverse transcription and dsDNA synthesis was terminated by incubating at 65°C for 15 min. The transcription master mix was prepared following the manufacturer's protocol (4X Transcription buffer, 0.1M DTT, NTP mix, 50% PEG, RNase-Out, Inorganic pyrophosphatase, T7-RNA polymerase, and Cyanine 3-CTP). Transcription of dsDNA was performed by adding the transcription master mix to the dsDNA reaction samples and incubating at 40°C for 2 h. Amplified and labeled cRNA was purified on cRNA Cleanup Module (Agilent Technology) according to the manufacturer's protocol. Labeled cRNA target was quantified using ND-1000 spectrophotometer (NanoDrop Technologies, Inc). After checking labeling efficiency, fragmentation of cRNA was performed by adding 10X blocking agent and 25X fragmentation buffer and incubating at 60°C for 30 min. The fragmented cRNA was resuspended with 2X hybridization buffer and directly pipetted onto assembled Agilent's Mouse Oligo Microarray (44K). The arrays were then hybridized at 65°C for 17 h using Agilent Hybridization oven (Agilent Technology). The hybridized microarrays were washed according to the manufacturer's washing protocol (Agilent Technology). The hybridized images were scanned using Agilent's DNA microarray scanner and quantified with Feature Extraction Software (Agilent Technology). All data normalization and selection of fold-changed genes were performed using GeneSpringGX 7.3 (Agilent Technology). The averages of normalized ratios were calculated by dividing the average of normalized signal channel intensity by the average of normalized control channel intensity. Functional annotation of genes was performed according to Gene OntologyTM Consortium (http://www.geneontology.org/index.shtml) by GeneSpringGX 7.3. Gene classification was based on searches done by BioCarta (http://www.biocarta.com/), GenMAPP (http://www.genmapp.org/), DAVID (http://david.abcc.ncifcrf.gov/), and Medline databases

(http://www.ncbi.nlm.nih.gov/). The Ingenuity Pathway Analysis (IPA) tool (QIAGEN) was used to test whether its pre-defined knowledge of Smad2 and 3 pathways matched with the upregulated/downregulated genes in our microarray dataset calculated based on the values of PCBs against Flk1$^+$ MPCs. Each Agilent probeset which met a $P < 0.05$ threshold and a fold change of ≥ 2 were mapped to its corresponding gene object in the Ingenuity Pathways Knowledge Base. Original data are available in the National Center for Biotechnology Information's Gene Expression Omnibus (accession number GSE65791).

Chromatin immunoprecipitation (ChIP) analysis

ChIP assay was performed with EZ ChIP kit (Millipore-Upstate) according to the manufacturer's manual. Briefly, approximately 1×10^7 cells were incubated in cell culture medium containing 1% formaldehyde at RT for 10 min and quenched by the addition of glycine for 5 min at RT. Cells were washed twice in ice-cold PBS and resuspended in 1 ml of SDS lysis buffer containing protease inhibitor cocktail II. After aliquoting each sample into 300 μl, cross-linked chromatin was fragmented by sonication and pre-cleared with protein G agarose at 4°C for 1 h. Chromatin was then incubated with each antibodies overnight at 4°C. Antibodies (Millipore-Upstate) used for the ChIP assay were as follows: Anti-monomethyl-H3K4 (Millipore, #07-436), anti-trimethyl-H3K4 (Millipore, #04-745), anti-trimethyl-H3K27 (Millipore, #07-449) and anti-acetyl-H3K9 (Millipore, #06-942). Immunocomplexes were incubated with protein G agarose at 4°C for 1 h, and the immunoprecipitates were washed once with low salt wash buffer, once with high salt wash buffer, once with LiCl wash buffer, and twice with TE buffer. Samples were resuspended in elution buffer and incubated at 65°C overnight. DNA samples were isolated using spin columns. Quantitative PCR was carried out on a CFX connect Real-time system (Bio-Rad). The primers are shown in Supplementary Table 2. ChIP-qPCR results were calculated using the ΔΔCt method. The Ct value of the respective ChIP fraction was normalized against the Ct value of the input DNA fraction. Then, the Ct value of the ChIP fraction was again normalized to the Ct value of the IgG control. Fold enrichment of immunoprecipitation was calculated by $2^{-\Delta\Delta Ct}$.

Bisulfite sequencing

The bisulfite sequencing was performed with EZ DNA Methylation-Gold kit (Zymo) according to the manufacturer's manual. Briefly, respective DNA samples were mixed with CT conversion reagent and incubated in a thermal cycler with the following steps: 98°C for 10 min, 64°C for 2.5 h, and 4°C storage up to 20 h. After binding to IC columns, DNA samples were desulphonated at RT for 15 min. Desulphonated DNA samples were eluted and used

for further PCR. Primers that were used for PCR are shown in Supplemental Table 2. PCR products were purified with MEGA-spin agarose gel extraction kit (Intron biotechnology) and ligated into pGEM T-easy vector (Promega). Methylation status was analyzed by sequencing the data and represented as a closed circle for methylated CpG and an open circle for unmethylated CpG.

Statistical analyses

Values are presented as mean ± standard deviation (SD). For continuous data, statistical significance was determined with the Mann-Whitney *U* test between 2 groups and the Kruskal-Wallis test followed by Tukey's honest significant difference (HSD) test with ranks or multiple-group comparison. Statistical analysis was performed with SAS 9.4 (SAS Institute Inc). Statistical significance was set at $p < 0.05$ or 0.01.

SUPPLEMENTARY REFERENCES

1 Yamashita, J. K. *et al.* Prospective identification of cardiac progenitors by a novel single cell-based cardiomyocyte induction. *FASEB journal : official publication of the Federation of American Societies for Experimental Biology* **19**, 1534-1536, doi:10.1096/fj.04-3540fje (2005).

2 Hirai, H. *et al.* Hemogenic and nonhemogenic endothelium can be distinguished by the activity of fetal liver kinase (Flk)-1 promoter/enhancer during mouse embryogenesis. *Blood* **101**, 886-893, doi:10.1182/blood-2002-02-0655 (2003).

3 Kodama, H., Nose, M., Niida, S., Nishikawa, S. & Nishikawa, S. I. Involvement of the C-Kit Receptor in the Adhesion of Hematopoietic Stem-Cells to Stromal Cells. *Exp Hematol* **22**, 979-984 (1994).

4 Cho, H. J. *et al.* Induction of pluripotent stem cells from adult somatic cells by protein-based reprogramming without genetic manipulation. *Blood* **116**, 386-395, doi:10.1182/blood-2010-02-269589 (2010).

5 Takahashi, K. *et al.* Induction of pluripotent stem cells from adult human fibroblasts by defined factors. *Cell* **131**, 861-872, doi:10.1016/j.cell.2007.11.019 (2007).

6 Uosaki, H. *et al.* Efficient and Scalable Purification of Cardiomyocytes from Human Embryonic and Induced Pluripotent Stem Cells by VCAM1 Surface Expression. *Plos One* **6**, doi:10.1371/journal.pone.0023657 (2011).
